# Implementation of Parenting Programs in Real-World Community Settings: A Scoping Review

**DOI:** 10.1007/s10567-023-00465-0

**Published:** 2023-12-07

**Authors:** Rita Pinto, Catarina Canário, Patty Leijten, Maria José Rodrigo, Orlanda Cruz

**Affiliations:** 1 Faculty of Psychology and Education Sciences, University of Porto, Porto, Portugal; 2 Research Institute for Child Development and Education, University of Amsterdam, Amsterdam, The Netherlands; 3 Department of Developmental Psychology and Education, Faculty of Psychology, University of La Laguna, Santa Cruz de Tenerife, Spain

**Keywords:** Parenting programs, Implementation, Real-world community settings, Scoping review

## Abstract

Implementing parenting programs in real-world community settings is fundamental to making effective programs widely available and consequently improving the lives of children and their families. Despite the literature acknowledging that the high-quality implementation of parenting programs is particularly challenging in real-world community settings, little is known about how the programs are implemented in these settings. This scoping review followed the methodological framework described by the Joanna Briggs Institute to map evidence on how evidence-based parenting programs have been implemented under real-world conditions. A systematic search of 12 scientific databases, gray literature, and the reference lists of the included studies identified 1918 records, of which 145 were included in the review. Fifty-three parenting programs were identified in studies documenting implementation in real-world community settings worldwide. Most studies included families in psychosocial risk engaged with family-support agencies. The qualitative synthesis identified several implementation outcomes, adaptations, barriers, and facilitators. Most studies reported a maximum of two implementation outcomes, mainly fidelity and acceptability. Providers frequently made adaptations, mainly to bring down barriers and to tailor the program to improve its fit. Findings highlight the need for a more detailed description of the implementation of programs, with greater consistency in terminology, operationalization, and measurement of implementation outcomes across studies. This will promote a more transparent, consistent, and accurate evaluation and reporting of implementation and increase the public health impact of parenting programs. Future studies should also assess the impact of adaptations and the cost-effectiveness and sustainability of programs in real-world community settings.

**Supplementary Information:**

The online version contains supplementary material available at 10.1007/s10567-023-00465-0.

## Introduction

The primary role of the family in promoting child’s everyday well-being and long-term development is well established. Children are at greater risk for emotional and behavioral problems if they are raised in family environments that are conflicted and unpredictable (Szepsenwol et al., 2022). Children benefit most from their family when parents are warm, responsive, and supportive; when they monitor and discipline appropriately (Kuppens & Ceulemans, 2019). Therefore, consensus has been reached on the need to support parents to fulfill the right of children to grow up in healthy family contexts, through providing parenting programs with a strong evidence base (Doyle et al., 2023; Lansford et al., 2022; Prinz, 2019; Rodrigo, 2016). Parenting programs can improve parenting practices and reduce family-related risk factors associated with child maltreatment and the development of emotional and behavioral difficulties in children and adolescents (Prinz, 2019; Vlahovicova et al., 2017; World Health Organization, 2022).

The effectiveness of parenting programs strongly depends on the quality of their implementation (Baumann et al., 2016; Durlak, 2015). This raises the question of how parenting programs in the community are being implemented to support parents and children (Lansford et al., 2022). In this review, we systematically searched the literature for studies documenting parenting program’s implementation in real-world community settings. We adopted a scoping review approach (Peters et al., 2020) to map the available evidence on this topic.

Evidence-based parenting programs are theoretically driven and empirically validated, with contents, activities, and methodology described and structured in a manual (Rodrigo, 2016). These programs are provided to improve parents’ knowledge, skills, and confidence to best support their children’s development; to reduce children’s emotional and behavioral difficulties and parental stress; and to prevent child maltreatment and optimize children’s development (Barlow & Coren, 2018; Prinz, 2019). Prevention scientists distinguish between programs’ efficacy and effectiveness. Efficacy relates to the effects of a program under ideal implementation conditions, whereas effectiveness refers to the effects of a program implemented under real-world conditions (Flay et al., 2005; Gottfredson et al., 2015).

High-quality implementation of programs is particularly challenging under real-world conditions and the transferability of parenting programs to real-world community settings is scarcely understood (Durlak, 2015; Lansford et al., 2022; Powers et al., 2015). Quality of implementation refers to a specific set of activities designed to put into practice an activity or program, incorporating it into real-world community settings to benefit children, families, services, and communities (Fixsen et al., 2009). Although the delivery of parenting programs in real-world community settings has been progressively encouraged, the inability to appropriately implement them can limit the potential for families to benefit from advances (Rodrigo, 2016). Indeed, many research findings are not routinely translated into practice, and many authors emphasized that this research-to-practice gap is due to the lack of attention paid to the implementation process (Durlak & DuPre, 2008; Lansford et al., 2022; Miller et al., 2012; Premachandra & Lewis, 2022). When the implementation is not properly addressed, inadequate outcomes may be mistakenly attributed to the program theory of change, rather than the omission of core program functions (i.e., the active ingredients of a program that are primarily associated with its effectiveness) (Durlak & DuPre, 2008; Kirk et al., 2021).

There is an increasing recognition that discriminating the intervention outcomes from the implementation outcomes is critical (Fixsen et al., 2009; Proctor et al., 2011). Implementation outcomes are the effects of deliberate and purposive actions to implement new programs, practices, and services (Proctor et al., 2011), and they can be measured in terms of attitudes, opinions, intentions, or behaviors (The Centre for Effective Services, 2022). A myriad of implementation outcomes is identified in numerous frameworks. Two most frequently applied outcomes-focused implementation frameworks are the Reach, Effectiveness, Adoption, Implementation, and Maintenance Framework (RE-AIM; Glasgow et al., 1999, 2019) and the Implementation Outcomes Framework (Proctor et al., 2011). A recent study compared both frameworks and found substantial overlap in the implementation outcomes proposed by both frameworks (Reilly et al., 2020). Based on Reilly et al. (2020), we selected the framework proposed by Proctor et al. (2011) because it is more comprehensive, covering both the outcomes presented in RE-AIM and additional outcomes (e.g., acceptability). Furthermore, the framework by Proctor et al. (2011), despite being published a decade ago, continues to be frequently used in the literature (e.g., Khadjesari et al., 2020; Mettert et al., 2020; Xu et al., 2023).The proposed taxonomy in the Proctor’s framework includes eight implementation outcomes: (1) acceptability, (2) adoption, (3) appropriateness, (4) cost, (5) feasibility, (6) fidelity, (7) penetration, and (8) sustainability (Table 1).

**Table 1:** Taxonomy of eight implementation outcomes (adapted from Proctor et al., 2011)

Implementation outcome	Description
Acceptability	The perception among implementation stakeholders that a given program is agreeable or satisfactory. Other terms used in literature are related to the satisfaction with various aspects of the program (e.g., content, complexity, and delivery)
Adoption	The intention or initial decision to employ the program. Adoption also may be referred to as uptake
Appropriateness	The perceived fit, relevance, or compatibility of the program for a given practice setting, provider, or consumer; and/or perceived fit of the program to address a particular issue
Cost	The cost of implementing the program, and any costs required to support its implementation
Feasibility	The extent to which the program can be successfully used or carried out within a given setting
Fidelity	The degree to which the program was implemented as it was prescribed in the original protocol
Penetration	The integration of the program within a service setting and its subsystems. From a service system perspective, the construct is similar to ‘‘Reach’’ in the RE-AIM framework
Sustainability	The extent to which a newly implemented program is maintained or institutionalized within a service setting’s ongoing operations. The construct is similar to “Maintenance’’ in the RE-AIM framework

Typically, research in real-world settings progresses from effectiveness studies (does an intervention work under real-world conditions?) to implementation studies (how is an intervention delivered in real-world community settings?) (Shepardson & Polaha, 2023). However, most researchers agree that effectiveness and implementation research should not be separate and sequential, and doing so overlooks complexity and slows the transferability of research findings into routine practice (Curran et al., 2022; Landes et al., 2019; Shepardson & Polaha, 2023). Curran et al. (2012) introduced effectiveness-implementation hybrid studies, which combine the examination of both effectiveness and implementation outcomes and intend to answer, within the same study, questions regarding whether the intervention works and how to best implement it. Hybrid studies exist on a continuum, varying from whether their primary focus and emphasis is on either effectiveness or implementation outcomes (Curran et al., 2012, 2022). In a hybrid type 1 study, the primary aim is to determine intervention effectiveness while exploring implementation issues (e.g., describe barriers and facilitators; ask participants regarding implementation experience). A hybrid type 2 study involves the simultaneous testing of both intervention effectiveness and implementation. In a hybrid type 3 study, the main aim is evaluating the implementation of an intervention (e.g., testing the implementation activities used to deliver the intervention and the impact of those activities on an intervention’s adoption, fidelity, and other implementation outcomes) while also gathering information on intervention effectiveness.

Integrating the examination of program implementation and effectiveness in one study can yield valuable insights into possible explanations for observed effectiveness or lack thereof. Attending to implementation immediately at the start of evaluating program effectiveness (rather than first establishing that an intervention is effective) can, therefore, help refine interventions based on the feedback of families, practitioners, and stakeholders (Curran et al., 2022; Shepardson & Polaha, 2023).

Since the implementation outcomes are considered the pre-conditions for achieving effective program outcomes, the field benefits from studies not only carefully evaluating them in the implementation process but also reporting on this in their papers (Lengnick-Hall et al., 2022; Pinnock et al., 2017; The Centre for Effective Services, 2022). However, Lengnick-Hall et al. (2022) found several problems in studies when reporting the implementation outcomes, namely lack of clarity about the specific outcome being addressed, inconsistent terminology, and lack of specificity around how the outcome was measured. These authors proposed six recommendations to prevent reporting problems in future work: (1) to describe each implementation outcome, provide the definition that the study will use and ensure consistent use of terms and definitions across manuscript sections; (2) to state how each implementation outcome will be or was analyzed relative to other constructs; (3) to specify what each implementation outcome will be measured in relation to; (4) to report who provided data, the level at which data were collected for each implementation outcome, and what type of data was or will be used to assess each implementation outcome; (5) to state the number of time points and the frequency at which each outcome was or will be measured; and finally, (6) to specify the unit of analysis and unit of observation for each implementation outcome.

Reporting program implementation is recognized as fundamental for programs’ adoption, replication, and scale-up (Paulsell et al., 2014). Despite recommendations that the implementation process should be described in sufficient detail such that independent observers can detect the presence of the set of activities related to implementation (Fixsen et al., 2009; Pinnock et al., 2017), most of the information about implementation in empirical studies are typically underreported and remains unknown (Gold et al., 2016; Hidalgo et al., 2016; Pinnock et al., 2017). The seminal literature review from Fixsen et al. (2009) pointed out that most studies reporting effectiveness trials do not describe information relevant to implementation. Bull et al. (2003) provided a literature review of worksite health behavior interventions and found that implementation data were reported only in 12.5% of the studies. A meta-analysis of prevention programs found that 68.5% of programs were presented without sufficient detail to allow replication (Domitrovich & Greenberg, 2000). Premachandra and Lewis (2022) found in their scoping review of 56 psychological intervention evaluations that authors reported, on average, 36% of the implementation information recommended by implementation scientists. Implementation factors are often taken into consideration (e.g., allocating resources to maintain high levels of intervention fidelity and using techniques to enhance the adoption and feasibility), but manuscript submission page limits and differences in terminology often leave these factors underreported (Rudd et al., 2020).

An important factor to consider and report when implementing a program in real-world dynamics are the adaptations, i.e., changes made to the program, either in the program content or in the way the program is delivered (Chambers & Norton, 2016; Durlak, 2015; Wiltsey Stirman et al., 2019). The complex interplay between program characteristics and service system, organizational, and family characteristics can impact the need to adapt interventions at different levels (service system, organization, provider, and client levels) to implement the program effectively (Chambers & Norton, 2016; Dusenbury et al., 2005). In contrast with efficacy trials, studies developed under real-world conditions demand more flexibility between program fidelity and adaptations in the program’s delivery (Anyon et al., 2019). Studies highlighted that this flexibility, when aligned with core principles underlying the program model, improves the program’s fit and is critical to success (Anyon et al., 2019). Despite an increasing amount of literature recognizing that program adaptation is inevitable (Chambers & Norton, 2016; Durlak, 2015; Wiltsey Stirman et al., 2019), our knowledge about what, how, and when adaptations occur and how practitioners balance them with fidelity during the implementation process is limited (Rodrigo, 2016). Recently, Escoffery et al. (2018) conducted a systematic review on adaptations of evidence-based public health interventions and found that the most frequently mentioned type of adaptations were content modifications, with the common reason to make the program relevant to a particular culture, new population, and setting. However, the authors did not review gray literature, and the results are mainly focused on HIV/AIDS, mental health, substance abuse, and chronic illnesses.

Other important factors to consider and report are barriers and facilitators of implementation. For instance, some facilitators are: (1) to establish a supportive leadership and organizational culture; (2) to provide adequate staff training and supervision; (3) to establish an implementation team composed of members with diverse expertise and skills responsible for driving and supervising the implementation process; and (4) to develop the implementation plan, which outlines key information required to achieve the desired outcomes from implementing the program (e.g., the resources needed and those already available; as well as the methods for the program’s monitoring and evaluation) (Barton & Akin, 2022; Metz et al., 2015; The Centre for Effective Services, 2022). Better knowledge of the barriers and facilitators will inform other implementers about what to avoid and what factors to replicate to achieve favorable results.

Even though implementation science has received growing interest from parenting program scholars, practitioners, funders, and decision-makers (Villalobos Dintrans et al., 2019), limited research has mapped issues in this area (Norton et al., 2017). Hoagwood et al. (2010) published a comprehensive review of 50 structured family-support programs for caregivers of children with identified mental health needs, synthesizing results on where the studies were conducted and their characteristics. In this work, the authors highlighted the need for greater attention on how family-support programs are delivered. Syntheses of research about implementation in the real world are recognized as essential not simply because they facilitate the identification of what works, with what population, and how in diverse contexts, but also because they bring to light the barriers and facilitators to the adoption of parenting programs in real-world community settings (Villalobos Dintrans et al., 2019; Wyborn et al., 2018). As parents cannot benefit from interventions they do not receive, it is also essential to know what parenting programs they receive (Fixsen et al., 2009). Such an advance would facilitate the spread of high-value, effective, and sustainable interventions in real-world community settings.

Research syntheses on the implementation of parenting programs under real-world conditions generate knowledge about what happens when programs are brought into new contexts (Lendrum & Humphrey, 2012). This understanding assists professionals in selecting and implementing these programs, thereby improving services and outcomes for children and their families. The present scoping review on the implementation of parenting programs in diverse community settings supports replication and increases the speed at which practitioners can use empirical findings (Lengnick-Hall et al., 2022). The proliferation of parenting programs implemented in real-world community settings has created new complexities for researchers, stakeholders, policymakers, and practitioners. Therefore, by synthesizing and integrating learnings across primary research studies, our scoping review will contribute to managing the proliferation of research knowledge in this field. This scoping review generates real-world evidence (i.e., evidence derived from the synthesis of real-world data), which will help to bridge the implementation gap between research evidence and its application in practice, providing useful information that helps to understand how parenting programs proven efficacious in controlled research settings can be effectively implemented in real-world practice.

In sum, the aim of this scoping review is to establish a comprehensive understanding of how parenting programs are implemented under real-world conditions, providing a map of available evidence and identifying knowledge gaps.

## Methods

This scoping review followed the Preferred Reporting Items for Systematic Reviews and Meta-Analysis extension for Scoping Reviews (PRISMA-ScR; Tricco et al., 2018). In addition, we used the guidelines set out by the JBI (formerly known as the Joanna Briggs Institute) for (1) identifying research questions; (2) identifying relevant studies; (3) study selection; (4) data charting; and (5) collating, summarizing, and reporting results (Peters et al., 2020).

We prospectively registered this scoping review at the Open Science Framework (available at: osf.io/4fydu) and published our study protocol (Pinto et al., 2021) before the database search. There are no differences between what was preregistered and what is presented in this scoping review. However, in the eligibility phase of the studies, we had to discuss what would be considered an evidence-based parenting program, as we realized that the extent of evidence for programs varied across studies. In order to map the field as comprehensively as possible, we decided to adopt an inclusive approach. This means we considered evidence-based parenting programs as all manualized parenting programs that have a clearly defined theoretical model, for which any level of initial evidence had been established for desired effects on improving parenting practices and reducing family-related risk factors associated with the development of emotional and behavioral problems in children and adolescents (Durlak & DuPre, 2008; Rodrigo, 2016).

### Identifying the Research Questions

The general research question that guided this scoping review was: How have evidence-based parenting programs been implemented under real-world conditions? To establish a comprehensive understanding of this topic, we formulated five specific research questions: (a) What parenting programs have been identified in studies documenting implementation in real-world community settings? (b) What are the main characteristics of the parents and children receiving these programs? (c) In what settings are these programs offered (e.g., healthcare facilities, schools, social service agencies, faith centers such as churches)? (d) What implementation outcomes (Proctor et al., 2011) and other implementation-related variables are reported? (e) What implementation barriers and facilitators were encountered?

### Identifying Relevant Studies

We piloted a preliminary search strategy with the keywords implement*, evidence-based program*, parent*, community, and real-world on Academic Search Ultimate and Scopus. From the results obtained, we noticed that studies used not only the term “evidence-based program”, but also “evidence-based intervention” and “evidence-based practice”, we added these keywords. Our final search string was: (implement* AND (“evidence-based program*” OR “evidence-based intervention” OR “evidence-based practice”) AND parent* AND (community OR “real world”)).

We searched multiple databases of peer-reviewed literature (Academic Search Ultimate, APA PsycArticles, APA PsycBooks, APA PsycInfo, Education Source, ERIC, Fonte Académica, Psychology and Behavioral Sciences Collection, PubMed, Scopus, Sociology Source Ultimate, and Web of Science), as well as gray literature in ProQuest databases, and the reference lists of included studies. We did not use time limits or restricted field tags. The literature search was conducted from September 2021 to December 2021 and updated in February 2022.

### Study Selection

We defined the eligibility criteria based on the JBI Population—Concept—Context (PCC) mnemonic. Studies were eligible if: (1) the implemented program targeted parents of children aged 2–16 years (Population); (2) the program was delivered in a real-world community setting, defined as a setting of routine practice, not a research-controlled setting (Context); and (3) they were empirical studies of evidence-based parenting programs (as we defined them previously in this section), identifying the factors related to the implementation process (Concept). To be included, studies must report implementation outcome variables (Proctor et al., 2011) and/or other implementation-related variables (e.g., adaptations, barriers, and facilitators). We excluded studies only targeting foster/adoptive parents or children in extended family placements in order to uniformize, as much as possible, the population characteristics of the reviewed studies.

The study screening and selection were undertaken by the first two authors working independently. This process comprised three levels of screening: (1) title review, (2) abstract review, and (3) full-text review. In cases of uncertainty about the eligibility of a study at the title and abstract levels, the study was carried onto the next phase of the selection process. The third author solved disagreements in the full study screening.

### Data Charting

We developed a charting form on Microsoft Excel to record the key information of each source. As recommended by the JBI (Peters et al., 2020), the members of the review team became familiar with the source results and piloted the extraction form to ensure all relevant results were extracted. We extracted the following information from each study: (1) general study information (e.g., country where the study was conducted and research design); (2) participants sociodemographic characteristics (e.g., age, educational level, profession); (3) context characteristics (e.g., the organization that implemented the program); (4) program characteristics (e.g., program’s title, format and type of delivery); (5) implementation characteristics (e.g., implementation outcomes, adaptations, barriers, and facilitators). Study characteristics extracted are presented in Online Resource 1. Data extraction was performed independently by two authors. Inter-rater reliability was around 90% at abstract/title screening and around 70% at full-text screening, ensuring the accuracy of data collection. The disagreements were solved by a discussion with the third member of the review team to reach a consensus.

### Collating, Summarizing, and Reporting the Results

This review integrates quantitative and qualitative syntheses. First, we provide an overview of the included studies using descriptive analysis with a frequency count to answer the research questions about which parenting programs have documented implementation in real-world settings, the main characteristics of the parents, children, and providers participating in these programs, and the settings where these programs were delivered. Different programs within the same intervention’s system (e.g., Pathways Triple P, Standard Triple P, Triple P Online, and Self-Help Triple P) were counted as the same program. When the same program was culturally adapted and its name modified, we considered the name of the original program (e.g., the Early Risers program was culturally adapted for use in Mexico; the Strengthening Families Program was culturally adapted for use in Spain). We also combined multiple records of the same study (e.g., a thesis and a peer-reviewed paper reporting on the same study).

Regarding the research questions about what implementation outcomes and other implementation-related information were reported, and what implementation barriers and facilitators were encountered, we performed a qualitative synthesis classifying the results under main conceptual categories, as suggested by the JBI manual (Peters et al., 2020). These conceptual categories were discussed and refined by the research team.

Following a deductive approach, we synthesized the extracted data about implementation outcomes from the set of variables proposed by Proctor et al. (2011). We established three levels of reporting: mentioning, monitoring, and measuring. (1) Mentioning refers to whether the study stated the implementation outcome (e.g., the study discussed that practitioners strived towards implementing the program with fidelity). (2) Monitoring means that the study not only mentioned the implementation outcome but also used specific indicators to evaluate it (e.g., the study reported that it monitored the fidelity of implementation by asking practitioners to fill summary checklists at the end of each session; the study reported that program’s acceptability was assessed using the Evidence-Based Practice Attitudes Scale). (3) Measuring means that the study not only monitored the implementation outcome but also provided the results obtained (quantitative or qualitative) (e.g., the study reported that average fidelity to program content was 81%; the study reported the intervention’s high level of appropriateness).

To organize the results about the implementation barriers and facilitators, we grouped them into six categories using the six key implementation factors proposed by Akin et al. (2014): (1) client factors (e.g., the complexity of participants’ concerns, their perceptions of the program, resistance towards the intervention); (2) provider factors (i.e., individual providers characteristics such as their attitudes about adopting and using the program); (3) program factors (i.e., aspects of the program itself, such as its complexity, adaptability, and cost); (4) process factors (e.g., staff selection, training, coaching, and performance assessment); (5) organizational factors (e.g., administrators’ attitudes, leadership, organizational culture and climate); and (6) structural factors (i.e., those that occur at the system level including the broader environment in which organizations operate, such as workforce issues and interagency collaboration). All these factors have the potential to either hinder or facilitate program implementation.

## Results

### Included Studies

**Fig. 1:**
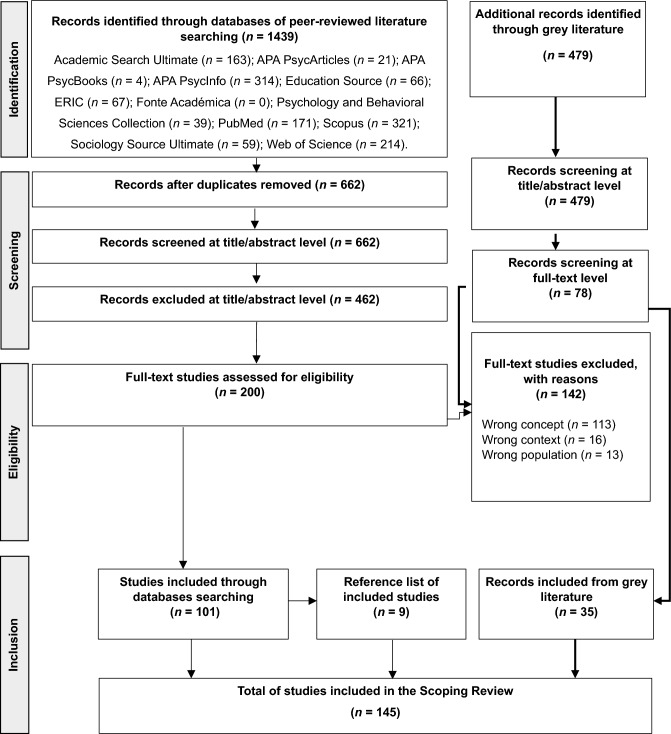
The PRISMA flow diagram for the scoping review process (adapted from Moher et al., 2009)

Our systematic literature search identified 1,918 records (see Fig. 1). Of these, 145 studies, 110 from peer-reviewed journals and 35 from the gray literature, were eligible for inclusion.

Studies were conducted in 21 different countries on five different continents. The majority were conducted in the United States of America (*n* = 92), followed by Canada (*n* = 12), Australia (*n* = 7), Spain (*n* = 6), Norway (*n* = 4), South Africa (*n* = 4), and the United Kingdom (*n* = 4). The remaining studies were from Belgium, Brazil, China, Germany, Iceland, India, Ireland, Israel, Kenya, Lebanon, Mexico, Nigeria, Romania, and Thailand. Our Online Resource 1 provides a summary of the characteristics of the studies included in this review (e.g., the programs that were implemented and the implementation outcomes that were reported).

### What Parenting Programs Have Been Identified in Studies Documenting Implementation in Real-World Community Settings?

Implementation of 53 different programs was reported. These parenting programs are mostly based on social learning theory principles, designed to improve parent–child relationship quality and behavior management in order to reduce disruptive child behavior. Most studies reported on the implementation of the Triple P—Positive Parenting Program (*n* = 31), Parent–Child Interaction Therapy (*n* = 24), Strengthening Families (*n* = 15), and Incredible Years (*n* = 12).

### What are the Main Characteristics of the Families who Received These Programs and of the Providers who Implemented Them?

Few studies described at least some characteristics of the programs’ participants. In terms of gender, 64 studies (44%) reported on parent gender, stating that parents were primarily mothers (on average 80%). In the 39 studies (27%) that reported on child’s gender, boys were overrepresented (on average 60%). The child’s age was reported in 56% of the studies (*n* = 81), of which 81% (*n* = 66) integrated pre- and school-aged children (2–12 years old). Less studies integrated adolescents (*n* = 18).

In terms of socioeconomic status, studies that reported on parent educational level (28%, *n* = 41) stated that most parents attained a high school education. In line with this, of the 54 studies that provided information on family income and occupational status, families were predominantly socioeconomically disadvantaged. As described in 27% of the included studies (*n* = 39), most children lived in two-parent households. In the included studies, families were engaged with child mental health or social services due to mental and behavioral concerns at family, parent, and child levels (e.g., children exhibiting disruptive behaviors, identification of child abuse and neglect, poverty, parental substance abuse, mental health treatment, family conflict, migrant and refugee families).

In the 52 studies that reported on the gender of providers, they were primarily women (on average 87%). The average age of providers reported in 25 studies was 40 years old. Program providers were typically employed at the organizations delivering the programs as part of routine practice. Their academic backgrounds were diverse, coming from the social, health, and educational sectors. Social workers, psychologists, and nurses were in the lead, followed by counselors and teachers. Most of these professionals were described as having several years of working experience with families.

### In What Settings Were These Programs Offered?

Of the included studies, 41 (28%) stated that the program was delivered in a community setting without further specification. In the studies that specified the type of agency, most programs were offered in mental health (*n* = 29) and child welfare organizations (*n* = 26). In most of the remaining studies, the programs were delivered simultaneously in a variety of settings (e.g., social services, clinics, schools, non-profit organizations, and churches).

### What Implementation Outcomes Were Reported?

**Fig. 2:**
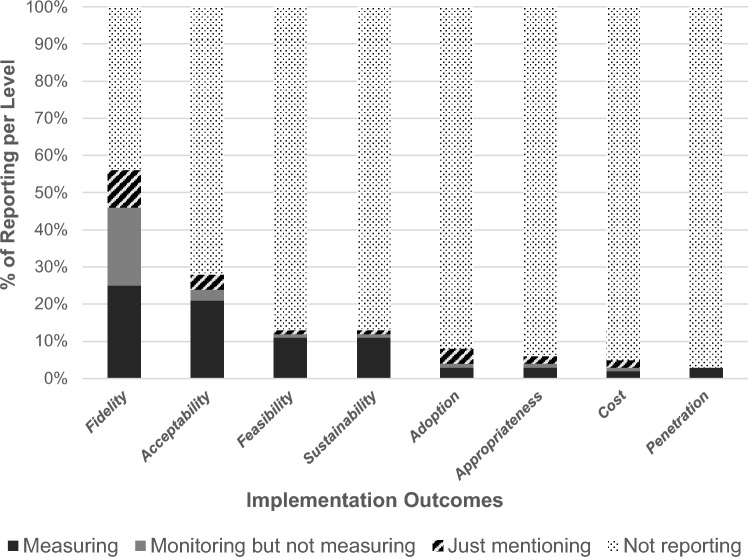
Studies’ percentage of reporting per level: mentioning, monitoring, and measuring

Most of the studies (*n* = 111) reported one or more implementation outcomes from the Proctor et al. (2011) framework (*M* = 1.68 [1, 6]; *SD* = 0.97). Almost a quarter of the studies (*n* = 34) did not report any implementation outcomes (see Fig. 2).

#### Fidelity

Fidelity was the most reported implementation outcome. 56% of the studies mentioned fidelity (*n* = 81), of which 81% (*n* = 66) monitored this implementation outcome employing the following methods: observation (through video or audio-recording; 41%, *n* = 27), self-report fidelity checklists (36%, *n* = 24), and both practitioners self-report in checklists and observation (20%, *n* = 13). Two studies did not report which method was used. Interestingly, five of the studies that used self-report checklists also measured fidelity from the perspective of those receiving the intervention (e.g., parents reported how often they had discussed each of the program’s topics with the provider). We noticed that most studies assessed fidelity in terms of how close the content and delivery methods conformed to the program’s manual (i.e., adherence to content). Of these, 23% (*n* = 15) also monitored how well the program was being delivered (i.e., the competence of delivery, such as the provider’s rapport, preparedness, and encouragement of participation). Of the 66 studies that used measures to monitor implementation fidelity, 55% (*n* = 36) reported the level of fidelity obtained. Fidelity ranged from 49.6 to 100%. On average, programs were implemented with a high level of fidelity (on average 82%). In four studies, fidelity was less than 60%.

#### Acceptability

The second most common implementation outcome in the studies was acceptability, being mentioned in 27% of the studies (*n* = 40). This includes five studies that, despite not using the term acceptability, measured parents’ and providers’ satisfaction with the program (e.g., its content and delivery) as an indicator of acceptability. Contrary to fidelity, which was defined relatively often, only seven studies reporting acceptability provided the operational definition for it. Of the 40 studies that mentioned acceptability, 82% (*n* = 33) monitored this implementation outcome, mostly through questionnaires and interviews on parents’ satisfaction with the program. Most of these studies (75%; *n* = 30) also measured acceptability, providing results on the level of acceptability obtained. Parents reported a high overall satisfaction with the program. For example, all parents rated the program as acceptable, with scores above 3 out of 4, in Dababnah and Parish (2014); and satisfaction with the program with a mean of 94.22 out of 100 in Lachman et al. (2016).

#### Sustainability

Sustainability was mentioned in 18 studies (12%). The studies were consistent in how they defined and monitored this implementation outcome. Sustainability was monitored in 94% of the studies (*n* = 17) in terms of continuation of program use (*n* = 14), maintenance of program results (*n* = 2), or both (*n* = 1). Most of these studies (94%, *n* = 16) also provided the results obtained. The percentage of providers that maintained the use of the program varied substantially across studies (e.g., in Frantz et al., 2015, it was 82%; in Hodge et al. (2017), it was 48.6% of providers). One study reported that approximately 70% of the providers had discontinued the delivery of the program (Barnett et al., 2021). In this case, the authors emphasized the need for someone within the organization who is certified to train colleagues in facilitating the program (commonly known as the train-the-trainer model) to overcome the staff turnover barrier.

#### Feasibility

Similar to sustainability, 18 studies mentioned feasibility. However, while sustainability is consistent in how it was defined and monitored, feasibility was described and assessed differently across studies. Feasibility was often examined in combination with other outcomes. For instance, one study (Lachman et al., 2016) defined feasibility as participant involvement, fidelity, and acceptability. Another (West et al., 2022) referred to feasibility as to the resources, training requirements, logistics, sustainability, and implementation challenges. Of the studies that mentioned feasibility, all but one showed evidence of having monitored this implementation outcome and almost all reported the data obtained on this. The nature of these data varied between studies. For example, one study (Dawson-Squibb et al., 2022) concluded that the program was feasible based on an examination of barriers and facilitators to implementation; another study (Yingling et al., 2020) inferred program feasibility from family completion and engagement rates.

#### Adoption

Substantially less frequently reported were adoption and appropriateness. Adoption was mentioned in 8% (*n* = 12) of the studies, including two studies that used the term uptake. In all but one study, adoption was monitored prior to program implementation, in contrast to the other implementation outcomes, which were all assessed during and after implementation. Of the studies that monitored adoption (*n* = 6), the one that did it post-implementation inferred it from intervention fidelity and family engagement rates; the remaining studies based their judgment of adoption on the perspectives of the providers and organization. Results were reported in 67% of the studies, with substantial variability in how adoption was measured. For instance, while Akin et al. (2016) stated that adoption was mainly successful across all five sites implementing the program; Asgary-Eden and Lee (2011) assessed adoption based on agencies’ readiness, openness, and resistance to implementation and presented ratings on a five-point scale.

#### Appropriateness

Appropriateness was mentioned in 6% of the studies (*n* = 9), including three studies that used the term perceived fit. Of the nine studies identified, six studies monitored this implementation outcome, and four studies also presented the results obtained. For instance, Lewis et al. (2016) assessed parents’ perceptions of the program’s appropriateness, reporting that most parents found the intervention to be useful and relevant; West et al. (2022) stated that six of the seven providers perceived that the program was a good fit and beneficial for Latino families. All studies defined appropriateness as the perceived fit of the program for parents and providers and monitored it through interviews with providers (*n* = 4) or parents (*n* = 2).

#### Cost and Penetration

The least reported implementation outcomes were cost and penetration. Cost was mentioned in 5% of the studies (*n* = 7). Of these, four studies reported that monitored the implementation costs and three also presented the costs obtained. These studies described the direct costs associated with the program’s implementation. For instance, Herschell et al. (2021) described payments to external trainers and payments to the provider agencies that compensated them for staff time away from service delivery to participate in training. In turn, Quetsch (2019) provided an analysis of cost incentives to increase retention and homework adherence and all costs incurred by the agency for irregular attendance. Any costs savings or long-term financial benefits were analyzed in these studies.

None of the studies mentioned the term penetration, but five studies (3%) monitored this implementation outcome using the term reach, which is a similar construct to penetration. These studies monitored the program’s reach and presented the results obtained (e.g., Hodge et al. (2017) described that the reach of program was 73.3% of the projection per provider; Polaha et al. (2018) described the number of families reached over 2 years of program’s implementation).

### What Other Implementation-Related Information Were Reported?

#### Adaptations

Parenting programs were adapted for real-world community settings in 40% of the studies (*n* = 58). Most studies (54 out of 58) reported changes in the way the program was delivered, either at the structural level (e.g., reordering the program’s sessions, lengthening or shortening the pace and timing of the program’s sessions) or procedural adaptations. Common procedural adaptations were, for example, language changes, including using simpler and more familiar terms for the families, and translation of materials when transferring the program from one country to another; and modifications in the examples used to match them with the realities of the families. Fewer studies (*n* = 31) reported adaptations in the program’s content. When this happened, it was mainly to add activities and topics believed to be helpful to the families or requested by them. Although the least frequently described adaptation, ten studies mentioned removing content, materials, and activities from the original program, with time-out being a strategy often removed. The main reason for the adaptations described was to improve the program’s fit for the context and families. For example, delivering sessions out of order to make them relevant to the family’s circumstances; adding pictures, or simplifying the program’s terms to make them more easily comprehended for parents with language barriers or low literacy. Three studies mentioned that providers made modifications to the program but did not specify these modifications.

#### Barriers and Facilitators

Most studies (74%, *n* = 108) provided an analysis of barriers and facilitators to the program’s implementation. The synthesis identified barriers and facilitators related to all the six key implementation factors proposed by Akin et al. (2014): influences at the client, provider, organization, program, process, and structural levels.

At the level of the client, the most reported barriers and facilitators were related to (1) logistical issues (e.g., scheduling sessions, transportation to the program’s local delivery, and childcare while the parent was participating in the program); and (2) parents’ engagement and their life circumstances, which could make it easier or harder for parents to get involved with the program (e.g., family income, single versus two-parent families, and the involvement or not with child protective services). Most studies emphasized the development of a strong therapeutic alliance with the provider, within a destigmatizing context, as key to parents’ engagement. At the provider level, the most reported barriers and facilitators were their workload, self-efficacy, and attitudes toward the program.

The program’s adaptability, the duration of sessions, and the level of language and terminology were the most barriers and facilitators reported at the program’s level. For instance, the programs being manualized and their adaptability were reported as allowing the provider to run sessions in a structured manner while using the flexibility to increase the program’s fit to the family’s needs. At the process level, most studies highlighted training and ongoing supervision as determinants for providers’ ability to deliver the program. Specifically, a time lag between training and the first in-field implementation was described as hampering the program’s implementation. How the program was introduced to families was also a process-related factor described as key to improving their willingness to participate, identifying what the parents consider attractive in the program, and highlighting these aspects to them in the family’s recruitment.

The support providers received from their leadership and the organization’s readiness (e.g., the availability of resources such as space and staff; the organization’s capacity to match the program with their existing services and practices) were reported as organization-related factors that could either facilitate or hinder the program’s implementation. At the structural level, funding and the number of families referred to these parenting programs were pointed out as crucial factors that could act as a barrier or facilitator to the program’s implementation. Specifically, some studies highlighted the need to improve inter-organizational networks and cross-system collaborations (e.g., finding key community partners to donate space) to ease the implementation of parenting programs in real-world community settings.

## Discussion

Understanding how parenting programs are implemented in real-world settings is essential for improving programs, family-support services, and outcomes for children and families. Despite recommendations that the implementation process should be adequately evaluated and reported, most of the information about the implementation process of parenting programs in real-world community settings remains underreported. This scoping review synthesized the reporting of implementation in parenting programs delivered in settings of routine practice with families. It illustrates that different parenting programs are implemented in diverse settings worldwide. However, we found a paucity of implementation details in the included studies, and when this information was reported, it occurred with high inconsistency in the terminology and measures used.

This scoping review synthesized evidence on the implementation of 53 parenting programs, implemented in 21 countries across the globe. Most of these programs are based primarily on social learning theory and cognitive–behavioral principles, focused on promoting child development (e.g., improving children’s adjustment across settings, reducing children’s emotional and behavioral problems, and preventing child maltreatment) through enhanced parenting skills and family environments. Parents participating were mostly mothers of pre- to school-aged children. Most families were engaged with family-support agencies (whether mental health institutions or social services) due to being in situations of psychosocial risk. In most of the studies, the parenting programs were implemented with a selective prevention effort of child maltreatment (i.e., families in which maltreatment has not already occurred but it was at high risk). Considering that previous studies have indicated the scarcity of parenting programs available in family-support services (Axford & Morpeth, 2013; Forgatch et al., 2013), this finding suggests that some progress has been made over the last decade, with more parenting programs implemented in settings of routine practice. The target population reached in most of these settings are at-risk families who suffer from multiple psychosocial stressors that make participation more difficult. Implementing parenting programs on a population level could be a promising approach to reach more families and normalize parents’ participation in these programs. For instance, the included study from Frantz et al. (2015) conducted a community-wide implementation of Triple P and described an increase of families reached over time.

Most studies (*n* = 94, 65%) reported a maximum of two implementation outcomes. Implementation outcomes are often difficult to assess due to the complexity of implementing parenting programs in real-world community settings, which may explain why they remain underreported. This finding is consistent with previous research specifying reporting issues of implementation outcomes (Lengnick-Hall et al., 2022). Fidelity was the most reported implementation outcome in the included studies, followed by acceptability. The remaining implementation outcomes were substantially less reported. Sustainability and feasibility had similar percentages of reporting, followed by adoption, appropriateness, cost, and penetration. Interestingly, in studies where the implementation outcomes were mentioned, they were almost always monitored, and the results obtained were presented. For example, of the nine studies that reported appropriateness, three just mentioned, two just monitored and four measured this implementation outcome. It suggests that when the studies paid attention to the implementation outcomes, they did it thoroughly (mentioning, monitoring, and measuring).

A small majority of the studies reported fidelity, which shows a good awareness of the importance of program fidelity. It is only by making an appropriate evaluation of the fidelity with which an intervention has been implemented that an assessment can be made of its contribution to the program’s outcomes (Ginsburg et al., 2021). Without addressing fidelity, it is uncertain that the changes observed are due to the influence of the program being delivered and not due to variability in its implementation (e.g., elements that may have been added or essential elements of the program that were omitted). Notable is the reported process of fidelity monitoring in some studies, reconciling the use of self-report tools (fidelity checklists) and independent observation (video- and audio-recordings). There are advantages and disadvantages of both methods (e.g., there is a risk of desirability bias when using self-report measures, but providers may also act differently if they know they are being recorded), thus combining both methods may be a strategy to obtain more accurate data. For example, in Lachman et al. (2016), video recordings of a random sample of four sessions per parent group were examined to verify the accuracy of self-report data. Independently of the method chosen, an essential factor is the content of the fidelity instrument. An adequate measure needs to capture behaviors and processes that are consistent with the program’s core functions, as well as it should assess both providers’ adherence to these core functions and their competence in delivering them. Most of the included studies that did monitor fidelity have tended to focus exclusively on adherence to the protocol with less attention given to the providers’ competence of implementation. Previous studies suggested that the overall quality of intervention delivery is dependent upon both adherence and competence and that assessments of each provide different information and should be included (Fairburn & Cooper, 2011).

Sustainability is a key implementation outcome and a priority topic in implementation research (Walugembe et al., 2019), but most of the included studies did not report it. When this occurs, our understanding of factors that influence the maintenance of parenting programs in real-world settings is jeopardized. Failure to sustain parenting programs in routine services means that the intended benefits are short-lived and that real losses are incurred on research investment, time, and resources. Therefore, evaluating the cost-effectiveness and sustainability of parenting programs when they are transferred to real-world community settings should also be of interest to future research.

The analysis of the studies included in this review showed that providers frequently made adaptations. Most adaptations were changes in the way the program was delivered (e.g., in structure and language), though some studies also reported changes to the program’s content (e.g., adding topics). When we compare the adaptations reported and the barriers described, we realize that these adaptations were mainly to bring down faced or predicted barriers, tailoring the program to improve its fit and engage parents. For instance, the program was delivered at the family home to address parents’ lack of transportation as a barrier to their attendance (Gomi, 2015); the program materials were simplified to decrease the risk of alienating parents (Zerón, 2017); the order of modules differed between participants and new topics were incorporated to satisfy the families’ needs and requests (Oppenheim-Weller & Zeira, 2018). Even though tension exists between fidelity and adaptations (Theobald et al., 2018), contemporary conceptualizations support that both can coexist. In fact, we found a high level of fidelity described in the included studies and a high reporting of adaptations. We recognize that fidelity and adaptation are connected and, likely previous studies highlighted, it is challenging to balance these dimensions when implementing a parenting program under real-world conditions (Pérez et al., 2016). Maintaining high implementation fidelity preserves the program's core elements that initially demonstrated effectiveness but with the risk of being a poor fit for the new population or setting. We encourage studies to assess and report the impact of adaptations (e.g., instances where an adaptation may help improve acceptability, but may decrease fidelity). Examining adaptations’ impact will provide greater insight into how adaptations function, including what adaptations have positive, negative, or neutral impact on both implementation and intervention outcomes (Kirk et al., 2020).

### Implications

The importance of parenting programs’ implementation in real-world community settings has been well documented, but achieving implementation quality is a complex and demanding process. However, this is possible, and we found studies that are examples of good practices when reporting implementation. For instance, Akin et al. (2016) and Mathias et al. (2022) examined the parenting program’s implementation and reported three implementation outcomes and adaptations, barriers, and facilitators. In Hodge et al. (2017), the authors assessed factors that are associated with program delivery and discussed five implementation outcomes. Other studies, even reporting less implementation outcomes, reported them in a complete way, not only monitoring but also presenting the results (e.g., Dawson-Squibb et al., 2022; Lachman et al., 2016; West et al., 2022). The attention paid to the implementation process, such as what happened in these studies, facilitates better real-world implementation of parenting programs in the future. It is also important to highlight the hybrid studies included in our review, with a dual focus on examining the intervention’s effectiveness and implementation (Herschell et al., 2021; Norton et al., 2021; Smith et al., 2015). This typology of studies should be encouraged as they inform about meaningful effectiveness-implementation interactions, which may speed up the translation of research findings into routine practice and improve the dissemination and implementation quality of parenting programs in real-world community settings (Curran et al., 2022; Landes et al., 2019; Shepardson & Polaha, 2023).

Key efforts were described in the included studies to improve the quality of the programs’ implementation, such as elaborating an implementation plan and establishing an implementation team (e.g., Ogden et al., 2005; Yingling et al., 2020). Proctor et al.’s taxonomy was a useful framework to guide the synthesis of key implementation outcomes in this review and it is encouraging to see that some of the included studies also used it to guide the assessment of implementation (e.g., Jackson et al., 2017; Lewis et al., 2016). A recommendation would be to provide more clarification of feasibility, as each study included in this review conceptualized this implementation outcome differently. Proctor et al. (2011) encouraged future studies to extend this taxonomy and include other concepts. In this sense, another recommendation would be to add program’s adaptability as an implementation outcome.

Due to the need for more consistency in terminology, operationalization, and measurement of implementation outcomes across the included studies, analyzing and synthesizing data from this review was challenging. Further efforts are needed to standardize the conceptualization of implementation outcomes. Over the years, several authors have independently developed conceptual models, frameworks, and guidelines regarding how implementation can be carried out, assessed, and reported effectively (e.g., Berkel et al., 2011; Damschroder et al., 2022; Fixsen et al., 2009; Pinnock et al., 2017; Proctor et al., 2011). Reaching sufficient consensus across these different tools (e.g., common language, definitions, foundations for measurement, and reporting) is essential to increase the capability of other researchers, program developers, and providers to take advantage of it. Adequate reporting of implementation is crucial not only because it contributes to shared knowledge and language among researchers. It also provides the information required by both agency leadership to consider which interventions to adopt in their settings and by professionals to determine how to implement the intervention (Premachandra & Lewis, 2022; Rudd et al., 2020). Improved reporting of implementation outcomes would also facilitate the inclusion of this information in future scoping and systematic reviews and meta-analyses (Rudd et al., 2020). Establishing reliable methods, tools, and indicators for the adequate measurement of the implementation outcomes should be the next step. Taken together, conceptualization uniformity and instruments reliability will promote a more transparent, consistent, and accurate evaluation and reporting of implementation and increase the public health impact of parenting programs.

### Strengths and Limitations of the Study

Following a strong scoping review process and adhering to established reporting guidelines enhanced the rigor of our review design and trustworthiness of our findings about the state of knowledge on the implementation of parenting programs in real-world community settings. This review mapped the evidence on the implementation of parenting programs in a broad range of settings and interventions, as well as synthesized the report of several implementation outcomes, which provides a more comprehensive understanding of this field. Another strength of this review is that we performed a comprehensive search of relevant studies to be included across a considerable number of databases, with no limitation on publication dates. This scoping review was based on a rigorous methodological framework previously published in its protocol (Pinto et al., 2021), following the principles of open science such as transparency and reproducibility. The results of the scoping review were presented following the PRISMA-ScR which allowed complete reporting. To achieve a comprehensive review, we used systematic search, screening, and eligibility procedures to synthesize not only peer-reviewed but also gray literature, which is one of the strengths of this scoping review.

In addition to these strengths, some limitations must be considered. The included studies derived from the implementation of parenting interventions with research support that may not be representative of community practice without researchers’ supervision. Looking at studies that examine agency-based data sources or perspectives of organization’s practitioners, supervisors, or administrators would likely provide a closer approximation of usual practice for examining the extent to which parenting programs are sustained by the organization resources following the research study. By following the criteria for a scientific and gray literature search made in international databases, we might have missed work from countries that may be underrepresented in the international literature, as well as other studies that are still ongoing and not yet published. In addition, to ensure comparability of the included studies, we excluded studies only targeting foster or adoptive parents, or children in extended family placements, not characterizing the evidence on implementation of the studies targeting these types of families.

## Conclusion

Documenting the implementation of parenting programs in real-world community settings builds a bridge between the controlled settings where programs are developed and the real-world community settings where they are delivered. This scoping review identified implementation reporting issues that, if addressed, will help enhance the scientific reporting quality and transparency of research. In addition, improving the reporting of implementation is essential to assist providers and host organizations in selecting and delivering the programs effectively. By increasing our understanding of how, why, and under what conditions a parenting program does or does not work, we maximize the chances that these programs will be effectively implemented to successfully benefit children and their families.

### Supplementary Information

Below is the link to the electronic supplementary material.

Supplementary file1 (PDF 1060 kb)

## Data Availability

This study was registered at the Open Science Framework (osf.io/4fydu) and its protocol is available at https://doi.org/10.1371/journal.pone.0256392
