# Supplementary material for: Implementation of Parenting Programs in Real-World Community Settings: A Scoping Review
Source: Clin Child Fam Psychol Rev. 2023 Dec 7;27(1):74–90. doi: 10.1007/s10567-023-00465-0 (PMC10920434; doi:10.1007/s10567-023-00465-0)
Supplement: Supplementary file 1 — Supplementary file1 (PDF 1060 kb) [file 10567_2023_465_MOESM1_ESM.pdf]

Implementation of parenting programs in real-world community settings: A scoping review

Clinical Child and Family Psychology Review

Rita Pinto[1], Catarina Canário, Patty Leijten, Maria José Rodrigo, & Orlanda Cruz

[1] Center for Psychology at University of Porto, Faculty of Psychology and Education Sciences, University of Porto, Portugal

[1]Corresponding Author Email: up201006360@edu.fpce.up.pt

Table 1.

Study, Program, and Context Characteristics: Country Where the Study was Conducted (if not Reported, Country of the Lead Author's Research Institute); Name of the Program Delivered and its Type of Delivery/Format; the Setting in Which the Program was Implemented.

Authors (year)	Country	Programs	Delivery/Format	Settings
Aalborg et al. (2010)	United States of America (USA)	Strengthening Families Program 10–14*<br>Family Matters**	Face-to-face/Group*<br>Telephone/Individual**	Health care settings/medical centres
Aarons et al. (2012)	USA	The Incredible Years (Basic IY)	Face-to-face/Group	Community-based organization providing residential substance abuse and mental health services
Abdala et al. (2020)	Brazil	Strengthening Families Program 10–14	Face-to-face/Group	Community-based setting
Akin et al. (2016a)	USA	Strengthening Families Program	Face-to-face/Group	Child welfare agencies

Authors (year)	Country	Programs	Delivery/Format	Settings
		Celebrating Families!		
Akin et al. (2016b)	USA	Strengthening Families Program<br>Celebrating Families!	Face-to-face/Group	Child welfare agencies
Allchin et al. (2022)	Australia	Let's Talk about Children	No info	Mental health agencies
Álvarez et al. (2016)	Spain	Growing Up Happily in the Family	Face-to-face/Group and individual	Local social services
Amador Buenabad et al. (2020)	Mexico	Leaving Traces on Your Life (Huellitas)<br>Parent Management Training - Oregon Model	Face-to-face/Group	School
Amarós-Martí et al. (2016)	Spain	Learning Together, Growing with Family	Face-to-face/Group	Non-profit organization
Arim et al. (2017)	Canada	Triple P	No info	Community-based setting
Arruabarrena et al. (2019)	Spain	SafeCare	Face-to-face/Individual	Child welfare agencies
Asgary-Eden & Lee (2011)	USA	Triple P	Face-to-face/Individual and Group	Community-based setting
Ballard (2017)	USA	Parent Management Training - Oregon Model	Face-to-face/Group	School
Bamberger (2016)	USA	Strengthening Families Program 10-14	Face-to-face/Group	Community-based setting
Barnett et al. (2014)	USA	Child–Parent Psychotherapy	No info	Mental health agencies
Barnett et al. (2021)	USA	Parent–Child Interaction Therapy	No info	Mental health agencies
Bartley (2017)	USA	Family Connections	No info	Community-based setting
Berkel et al. (2013)	USA	Strong African American Families	Face-to-face/Group	Community-based setting
Berkel et al. (2018)	USA	New Beginnings Program	Face-to-face/Group	Community-based setting
Bloomquist et al. (2016)	USA	Early Risers<br>Healthy Emotions Program	Face-to-face/Individual and Group	Mental health agencies
Brabson (2017)	USA	Parent-Child Interaction Therapy	No info	Community-based setting

Authors (year)	Country	Programs	Delivery/Format	Settings
Brabson et al. (2021)	USA	Parent-Child Interaction Therapy	No info	Clinics
Breitenstein et al. (2010)	USA	Chicago Parent Program	Face-to-face/Group	Childcare centers
Breitkreuz et al. (2011)	Canada	Triple P (levels 2 and 3)	No info	Family support centers
Butler (2019)	United Kingdom	Triple P Self-directed	Online, Self-directed/Individual	Community-based setting
Cantu et al. (2010)	USA	Strengthening Families Program 10–14	Face-to-face/Group	Faith-based organizations, social service agencies, the juvenile justice system and community substance-abuse prevention coalitions
Chaffin et al. (2016)	USA	SafeCare	Face-to-face/Individual	Child welfare agencies
Charest & Gagné (2019a)	Canada	Triple P	Face-to-face/No info	Child welfare services, primary social care services, community organizations, schools, and child daycare centers
Charest & Gagné (2019b)	Canada	Triple P (levels 2 to 5)	Face-to-face/Individual and Group	Health care agency, non-profit organization, school, and child daycare center
Chase et al. (2019)	USA	Parent-Child Interaction Therapy	No info	Mental health agencies
Chlebowski et al. (2018)	USA	AIM HI (An Individualized Mental Health Intervention for ASD)	No info	Mental health agencies
Christian et al. (2014)	USA	Parent-Child Interaction Therapy	No info	Mental health agencies
Clarke (2013)	United Kingdom	Triple P (levels 2 and 4)	Online and Face-to-face/Individual and Group	Health services
Clay (2018)	USA	Triple P	Face-to-face/No info	Local community agencies

Authors (year)	Country	Programs	Delivery/Format	Settings
Cluver et al. (2016)	South Africa	Sinovuyo Teen Caring Families (part of the Parenting for Lifelong Health initiative)	Face-to-face/Group	Child welfare agencies, church
Cooper et al. (2016)	USA	Strengthening Families Program 10–14	Face-to-face/Group	School buildings, religious, government, or some other public facility
Côté & Gagné (2020a)	Canada	Triple P	Face-to-face/No info	Childcare services, school, non-government organization, government primary care agency, and child welfare services
Côté & Gagné (2020b)	Canada	The Triple P (level 2)	Face-to-face/No info	School, childcare services, Non-governmental organizations<br>Governmental primary care agencies, and child welfare services
Dababnah & Parish (2014)	USA	The Incredible Years (IY Basic)	Face-to-face/Group	Community-based setting
Dawson-Squibb et al. (2022)	South Africa	Parent–Child Interaction Therapy	No info	Mental health agencies
De Paul et al. (2015)	Spain	SafeCare*<br>Incredible Years**	Face-to-face/Individual* and Group**	Child welfare agencies
Delawarde-Saïas et al. (2018)	Canada	Triple P (levels 2 to 5)	No info/Individual and Group	Child welfare agencies, school, childcare centers, non-profit organization
Denard (2017)	USA	Triple P	Face-to-face/No info	Child welfare agencies
Estrada et al. (2017)	USA	Familias Unidas	Face-to-face/Group	School
Estrada et al. (2018)	USA	eHealth Familias Unidas	Online/Individual	Parents' home or a community-based setting

Authors (year)	Country	Programs	Delivery/Format	Settings
Fang et al. (2022)	China	SREIA	Face-to-face/Individual and Group	Not-for-profit organization
Fawley-King et al. (2014)	Australia	Triple P	Face-to-face/Group	Churches, community centres, and other public spaces
Fowles et al. (2018)	USA	Parent-Child Interaction Therapy	No info	Parents' home, clinic
Frantz et al. (2015)	Germany	The Prevention Program for Externalizing Problem Behavior*<br>Triple P (levels 1 to 4)**	Face-to-face*/Group*<br>No info**	Schools, family services, youth-welfare services, clinics
Gagnon (2016)	USA	Guiding Good Choices	Face-to-face/Group	Schools
Gallitto et al. (2018)	Canada	SafeCare	Face-to-face/Individual	Child welfare agencies
Garcia et al. (2020)	USA	Triple P (levels 3 and 4)	Face-to-face/Individual and Group	Child welfare agencies
Glazemakers (2012)	Belgium	Triple P (all levels)	No info/Individual and Group	Child welfare agencies, schools
Gomi (2015)	USA	Parent Management Training - Oregon Model	Face-to-face/Individual	Child welfare agencies
Gordon & Cooper (2015)	USA	Parent–Child Interaction Therapy	Face-to-face/Individual	Clinic
Guastaferro et al. (2017)	USA	SafeCare<br>Parents as Teachers	Face-to-face/No info	Community-based setting
Haroz et al. (2019)	USA	Family Spirit	Face-to-face/Individual	Community-based setting
Heerman et al. (2018)	USA	Healthier Families Program	Face-to-face/Individual	Parks and recreation centers
Herschell et al. (2021)	USA	Parent-Child Interaction Therapy	Face-to-face/Individual	Mental health agencies
Hickey et al. (2018)	Ireland	Incredible Years	No info	Community-based setting
Hill et al. (2007)	USA	Strengthening Families Program 10–14	No info	Community-based setting

Authors (year)	Country	Programs	Delivery/Format	Settings
Hill et al. (2019)	USA	Strengthening Families Program 10-14	No info	Community-based setting
Hodge et al. (2017)	Australia	Triple P (Indigenous)	Face-to-face/Individual and Group	Child welfare agencies
Huebner et al. (2021)	USA	Sobriety Treatment and Recovery Teams	No info	Child welfare agencies
Ionutiu (2016)	Romania	Triple P (level 4)	Face-to-face/Group	Counseling and support center for parents and children
Jackson et al. (2017)	USA	Parent-Child Interaction Therapy	No info	Community-based setting
Kerns et al. (2017)	USA	Triple P (levels 3 and 4)	No info	Community-based setting
Klest (2014)	Norway	Parent Management Training - Oregon Model	No info	Child welfare agencies
Kumpfer et al. (2012)	Ireland	Strengthening Families Program 12–16	Face-to-face/Group	Interagency collaboration of national and county delinquency and substance abuse agencies
Kutash et al. (2012)	USA	Parent Connectors	Telephone calls/Individual	Community-based setting
Lachman et al. (2016)	South Africa	Sinovuyo Teen Caring Families (part of the Parenting for Lifelong Health initiative)	Face-to-face/Group	Non-profit organization
Lanier et al. (2011)	USA	Parent–Child Interaction Therapy	No info	Community-based setting
Lau et al. (2011)	USA	Incredible Years	Face-to-face/Group	Clinic, school
Leclair et al. (2017)	Canada	Incredible Years	Face-to-face/Group	Child welfare agencies
Lee et al. (2006)	USA	Early Risers	Face-to-face/Group	Community-based setting
Lengnick-Hall et al. (2019)	USA	SafeCare	Face-to-face/Individual	Child welfare agencies
Lent (2022)	USA	Parent-Child Interaction Therapy	Online/Individual and Group	Community-based setting
Lewis et al. (2016)	USA	Triple P (level 5)	Face-to-face/Individual	Child welfare agencies

Authors (year)	Country	Programs	Delivery/Format	Settings
Liebsack (2019)	USA	Parent-Child Interaction Therapy	No info	Mental health agencies
Lindsay et al. (2011)	United Kingdom	Incredible years<br>Triple P (level 4)<br>Strengthening Families, Strengthening Communities	Face-to-face/Group	Community centres, schools, clinics, and the premises of voluntary bodies
Love et al. (2016)	USA	Triple P Online Community	Online/Individual (self-directed)	Community-based setting
Lyon & Budd (2010)	USA	Parent Child Interaction Therapy	Face-to-face/No info	Mental health agencies
Lyons et al. (2022)	USA	Parent–Child Interaction Therapy	No info	Child welfare agencies
Margolis (2013)	Spain	Family Check-Up	No info	Schools
Mathias et al. (2022)	India	Parenting for Lifelong Health	Face-to-face/Group	Community-based setting
Matos et al. (2006)	USA	Parent–Child Interaction Therapy	Face-to-face/Individual	Community-based setting
Matsumoto et al. (2007)	Australia	Triple P	Telephone/Group and Telephone consultation	Community-based setting
Mauricio et al. (2019)	USA	Family Check-Up	Face-to-face/No info	Mental health agencies
McCoy et al. (2021)	Thailand	Parenting for Lifelong Health	Face-to-face/Group	Public health services
McDonald et al. (2012)	USA	Families and Schools Together	Face-to-face/Group	Refugee centre
McWilliams (2020)	USA	Incredible Years (IY Basic)	Face-to-face/Group	Community-based setting
Miller & Keating (2013)	Canada	Incredible Years	Face-to-face/Group	Community-based setting
Miller et al. (2010)	USA	Parents Matter!	No info	Community-based organizations, faith-based organizations, health departments, and local education agencies

Authors (year)	Country	Programs	Delivery/Format	Settings
Murry et al. (2018)	USA	Pathways for African American Success	Face-to-face and Technology-based/ Individual and Group	Community-based setting
Myers et al. (2020)	USA	Triple P	Face-to-face/No info	Child welfare agencies
Nelson et al. (2012)	USA	Parent-Child Interaction Therapy	No info	Mental health agencies
Nolan (2014)	USA	Triple P	No info	Community-based setting
Norton et al. (2021)	Australia	Confident Body, Confident Child	No info	Hospital
O'Connor et al. (2020)	USA	Healthy Dads Healthy Kids	Face-to-face/Group	Clinics
O'Donovan (2020)	Ireland	Strengthening Families Program	Face-to-face/Group	Community-based setting
Ofoha et al. (2018)	Nigeria	Parenting Education Program for Punishment Prevention	Face-to-face/Group	Community-based setting
Ogden et al. (2005)	Norway	Parent Management Training - Oregon Model	No info	Community-based setting
Ogden et al. (2009)	Norway	Parent Management Training - Oregon Model Multisystemic Therapy	No info	Child welfare, child mental health, and school-based agencies
Oppenheim-Weller & Zeira (2018)	Israel	SafeCare	Face-to-face/Individual	Child welfare agencies
Orte Socias et al. (2016)	Spain	Strengthening Families Program	Face-to-face/Group	Child welfare agencies
Osburn (2013)	USA	Incredible Years	Online (self-directed)/Individual	Community-based setting
Owens (2019)	USA	Triple P (levels 2 to 5)	No info	Community-based setting
Parker et al. (2020)	USA	Families Talking Together	Face-to-face/No info	Community-based setting
Parra Cardona et al. (2012)	USA	Parent Management Training - Oregon Model	Face-to-face/Group	Religious organization
Patras & Klest (2016)	Norway	Parent Management Training - Oregon Model	No info	Child welfare agencies

Authors (year)	Country	Programs	Delivery/Format	Settings
Patrick et al. (2008)	USA	Staying Connected with Your Teen (formely known as Parents Who Care)	Face-to-face/Group	Church
Pickard et al. (2016)	USA	ImPACT Online	Online (self-directed or with therapist assistance)/Individual	Community-based setting
Polaha et al. (2018)	USA	Family Check-Up	No info	Pediatric residency, training clinic
Porta et al. (2018)	USA	Early Risers	No info	Community-based setting
Poulsen et al. (2010)	Kenya	Parents Matter!	No info	Community-based setting
Puffer et al. (2017)	Thailand	Strengthening Families Program (adaptation: Happy Families)	Face-to-face/Group	International rescue committee
Quetsch (2019)	USA	Parent-Child Interaction Therapy	Face-to-face/No info	Mental health agencies
Ramos (2019)	USA	Parent-Based Coping Scaffolding Program	No info	Community-based setting
Romney et al. (2014)	USA	Triple P (levels 4 and 5)	Face-to-face/Group	Child welfare agencies<br>Mental health agencies
Roosa Ordway et al. (2018)	USA	Mothering from the Inside Out	Face-to-face/Individual	Mental health agencies
Rudo-Stern (2019)	USA	Family Check-Up	No info	Community-based setting
Saldana et al. (2021)	USA	Families Actively Improving Relationships	Face-to-face/No info	Child welfare agencies
Scudder & Herschell (2015)	USA	Parent–Child Interaction Therapy	No info	Community-based setting
Scudder et al. (2017)	USA	Parent-Child Interaction Therapy	No info	Community-based setting
Self-Brown et al. (2011)	USA	SafeCare	Face-to-face/Individual	Child welfare agencies
Shapiro & Charest (2020)	USA	Multisystemic Therapy<br>Parent Child Interaction Therapy<br>Alternatives for families: A cognitive behavioural therapy	No info/Individual and Group	Non-profit organization, state agency, school

Authors (year)	Country	Programs	Delivery/Format	Settings
		Triple P<br>Functional Family Therapy<br>Strengthening Families Program		
Shapiro et al. (2015)	USA	Triple P (levels 2 to 4)	Face-to-face/Individual and Group	School, non-profit organization, mental health agency, childcare, and social services
Sigmarsdóttir & Guðmundsdóttir (2013)	Iceland	Parent Management Training - Oregon Model	No info	Community-based setting
Sim et al. (2020)	Lebanon	Families Make the Difference	Face-to-face/Group	International rescue committee
Skale et al. (2020)	USA	Parent-Child Interaction Therapy<br>Child-Parent Psychotherapy	No info	Mental health agencies
Smith et al. (2015)	USA	Family Check-Up	No info	Mental health agencies
Stern et al. (2008)	Canada	Incredible Years	Face-to-face/Group	Early learning centers
Stokes (2014)	USA	Parent-Child Interaction Therapy	No info	Community-based setting
Suchman et al. (2020)	South Africa	Mothering from the Inside Out	Face-to-face/Group	Hospitals
Taylor et al. (2015)	Canada	Triple P (level 4)	Face-to-face/Individual and Group	Community-based setting
Thomson et al. (2014)	United Kingdom	Empowering Parents, Empowering Communities	Face-to-face/Group	Community-based setting
Turner & Sanders (2006)	Australia	Triple P (level 3)	Face-to-face/Individual	Community child health clinics
Weaver et al. (2011)	USA	Safe N′ Sound	No info	Clinic
Webster-Stratton et al. (2014)	USA	Incredible Years	Face-to-face/Group	Mental health agencies

Authors (year)	Country	Programs	Delivery/Format	Settings
West et al. (2022)	USA	Attachment and Biobehavioral Catch-up	Face-to-face/Individual	Non-profit organization
Whitaker et al. (2012)	USA	SafeCare	No info	Child welfare agencies
Whitaker et al. (2015)	USA	SafeCare	No info	Child welfare agencies
Woodfield et al. (2021)	Australia	Parent-Child Interaction Therapy	No info	Child welfare agencies, clinic, not-for-profit organization, schools
Yingling et al. (2020)	USA	Autism Parent Navigators	Face-to-face/Individual	Statewide parent support center
Zerón (2017)	USA	Parent Management Training - Oregon Model (adaptation: Parenting Through Change for Reunification)	Face-to-face/Group	Community-based setting

Note. When the same study reported the implementation of more than one program, and the type of program's delivery/format differs between these programs, we discriminated it using the wildcard character asterisk (*).

Table 2. *Studies Reporting of Implementation Outcomes, Adaptations, Barriers and Facilitators.*

Study	Implementation Outcomes								Adaptations	Barriers/Facilitators
	Acceptability	Adoption	Appropriateness	Cost	Feasibility	Fidelity	Penetration	Sustainability		
Aalborg et al. (2010)	-	-	-	-	-	R[3]	-	-	-	R
Aarons et al. (2012)	-	-	-	-	-	R[3]	-	-	R	R
Abdala et al. (2020)	-	R[1]	-	-	-	-	-	-	R	R
Akin et al. (2016a)	-	-	-	-	-	R[1]	-	-	R	R

Study	Implementation Outcomes								Adaptations	Barriers/Facilitators
	Acceptability	Adoption	Appropriateness	Cost	Feasibility	Fidelity	Penetration	Sustainability		
Akin et al. (2016b)	R[3]	R[3]	R[3]	-	-	R[1]	-	-	R	R
Allchin et al. (2022)	-	-	-	-	-	-	-	R[3]	-	-
Álvarez et al. (2016)	-	-	-	-	-	R[2]	-	-	R	R
Amador Buenabad et al. (2020)	-	-	-	-	-	R[2]	-	-	-	R
Amarós-Martí et al. (2016)	-	-	-	-	-	R[2]	-	-	-	R
Arim et al. (2017)	-	-	-	-	-	-	-	-	-	R
Arruabarrena et al. (2019)	-	-	-	-	R[3]	-	-	-	R	R
Asgary-Eden & Lee (2011)	-	R[3]	-	-	-	R[2]	-	-	-	R
Ballard (2017)	R[3]	-	-	-	R[3]	R[2]	-	-	R	-
Bamberger (2016)	-	-	-	-	-	-	-	-	-	R
Barnett et al. (2014)	-	-	-	-	-	R[2]	-	R[3]	-	R
Barnett et al. (2021)	-	-	-	-	-	-	-	R[3]	-	R
Bartley (2017)	-	-	-	-	-	R[3]	-	-	-	R
Berkel et al. (2013)	-	-	-	-	-	R[2]	-	-	R	R

Study	Implementation Outcomes								Adaptations	Barriers/Facilitators
	Acceptability	Adoption	Appropriateness	Cost	Feasibility	Fidelity	Penetration	Sustainability		
Berkel et al. (2018)	-	-	-	-	-	R[2]	-	-	-	-
Bloomquist et al. (2016)	-	-	-	-	-	R[3]	-	R[3]	R	-
Brabson et al. (2021)	-	-	-	-	-	-	-	R[2]	-	R
Brabson (2017)	-	-	-	-	-	-	-	-	-	R
Breitenstein et al. (2010)	-	-	-	-	-	R[3]	-	-	-	R
Breitkreuz et al. (2011)	-	-	-	-	-	-	-	-	R	R
Butler (2019)	-	-	-	-	-	-	-	-	-	R
Cantu et al. (2010)	-	-	-	-	-	R[3]	-	-	-	-
Chaffin et al. (2016)	-	-	-	-	-	R[2]	-	-	R	-
Charest & Gagné (2019a)	-	-	-	-	-	-	-	-	-	R
Charest & Gagné (2019b)	-	R[3]	-	-	-	-	R[3]	-	-	R
Chase et al. (2019)	-	-	-	-	-	R[2]	-	-	-	R
Chlebowski et al. (2018)	-	-	-	-	-	R[2]	-	-	-	R
Christian et al. (2014)	-	-	-	R[1]	-	-	-	R[3]	-	R

Study	Implementation Outcomes								Adaptations	Barriers/Facilitators
	Acceptability	Adoption	Appropriateness	Cost	Feasibility	Fidelity	Penetration	Sustainability		
Clarke (2013)	-	-	-	-	-	-	-	-	-	R
Clay (2018)	-	-	R[3]	-	-	-	-	-	-	R
Cluver et al. (2016)	R[3]	-	-	-	-	R[3]	-	-	-	R
Cooper et al. (2016)	-	-	-	-	-	-	-	-	R	-
Côté & Gagné (2020a)	-	-	-	-	-	-	-	-	-	R
Côté & Gagné (2020b)	-	-	-	-	-	-	-	-	R	R
Dababnah & Parish (2014)	R[3]	-	-	-	-	-	-	-	R	R
Dawson-Squibb et al. (2022)	R[3]	-	-	-	R[3]	R[3]	-	-	-	R
De Paul et al. (2015)	R[2]	-	-	R[3]	-	R[2]	-	-	R	R
Delawarde-Saïas et al. (2018)	-	-	-	-	-	-	-	-	-	R
Denard (2017)	-	-	-	-	-	-	-	-	-	R
Estrada et al. (2017)	-	-	-	-	-	R[3]	-	-	-	-
Estrada et al. (2018)	-	-	-	-	-	R[3]	-	-	R	-
Fang et al. (2022)	R[2]	-	-	-	-	R[3]	-	-	-	R

Study	Implementation Outcomes								Adaptations	Barriers/Facilitators
	Acceptability	Adoption	Appropriateness	Cost	Feasibility	Fidelity	Penetration	Sustainability		
Fawley-King et al. (2014)	-	-	-	-	-	R[1]	-	-	R	-
Fowles et al. (2018)	-	-	-	-	-	-	-	-	R	-
Frantz et al. (2015)	-	-	-	-	-	R[1]	R[3]	R[3]	-	R
Gagnon (2016)	-	-	-	-	-	R[3]	-	-	R	-
Gallitto et al. (2018)	-	-	-	-	-	R[2]	-	-	-	R
Garcia et al. (2020)	-	-	-	-	-	R[1]	-	-	-	R
Glazemakers (2012)	-	-	-	-	-	-	-	-	R	R
Gomi (2015)	-	-	-	-	-	-	-	-	R	R
Gordon & Cooper (2015)	-	-	-	-	-	R[2]	-	-	R	R
Guastaferro et al. (2017)	-	-	-	-	-	R[1]	-	-	R	R
Haroz et al. (2019)	-	-	-	-	-	-	-	-	R	R
Heerman et al. (2018)	-	R[3]	-	-	-	R[3]	-	R[3]	R	R
Herschell et al. (2021)	-	-	-	R[3]	-	-	-	R[3]	R	R
Hickey et al. (2018)	-	R[1]	R[1]	-	-	-	-	-	-	R

Study	Implementation Outcomes								Adaptations	Barriers/Facilitators
	Acceptability	Adoption	Appropriateness	Cost	Feasibility	Fidelity	Penetration	Sustainability		
Hill et al. (2007)	-	-	-	-	-	R[1]	-	-	R	-
Hill et al. (2019)	-	-	-	-	-	R[1]	-	-	-	-
Hodge et al. (2017)	R[1]	R[1]	-	-	-	R[3]	R[3]	R[3]	R	R
Huebner et al. (2021)	-	-	-	-	-	-	-	R[3]	-	-
Ionutiu (2016)	R[3]	-	-	-	-	-	-	-	R	R
Jackson et al. (2017)	R[3]	-	-	-	R[3]	-	-	-	-	-
Kerns et al. (2017)	-	-	-	-	-	-	-	-	-	R
Klest (2014)	-	-	-	-	-	-	-	-	-	R
Kumpfer et al. (2012)	-	-	-	-	-	R[2]	-	-	R	R
Kutash et al. (2012)	R[3]	-	-	-	-	R[2]	-	-	-	-
Lachman et al. (2016)	R[3]	-	R[2]	-	R[3]	R[3]	-	-	-	R
Lanier et al. (2011)	-	-	-	-	-	R[1]	-	-	-	-
Lau et al. (2011)	-	-	-	-	-	R[3]	-	-	-	R
Leclair et al. (2017)	-	-	-	-	-	R[3]	-	R[3]	R	R
Lee et al. (2006)	-	-	-	-	-	-	-	-	-	R

Study	Implementation Outcomes								Adaptations	Barriers/Facilitators
	Acceptability	Adoption	Appropriateness	Cost	Feasibility	Fidelity	Penetration	Sustainability		
Lengnick-Hall et al. (2019)	-	-	-	-	-	-	-	-	R	-
Lent (2022)	R^3	-	-	-	-	-	-	-	-	-
Lewis et al. (2016)	R^3	-	R^3	-	-	R^2	-	-	-	R
Liebsack (2019)	R^2	-	-	-	-	-	-	-	-	R
Lindsay et al. (2011)	-	-	-	-	-	R^2	-	-	-	-
Love et al. (2016)	R^3	-	-	-	R^3	-	-	-	-	R
Lyon & Budd (2010)	R^3	-	-	-	-	R^3	-	-	-	R
Lyons et al. (2022)	-	-	-	-	-	R^3	-	-	-	-
Margolis (2013)	-	R^2	-	-	R^2	R^3	-	-	R	-
Mathias et al. (2022)	R^3	-	-	-	R^3	R^3	-	-	R	R
Matos et al. (2006)	R^3	-	-	-	-	-	-	-	R	R
Matsumoto et al. (2007)	R^3	-	-	-	-	-	-	-	-	-
Mauricio et al. (2019)	-	-	-	-	-	R^2	-	-	-	-
McCoy et al. (2021)	-	-	-	-	R^3	R^3	-	-	R	R
McDonald et al. (2012)	-	-	-	-	-	R^2	-	-	R	R

Study	Implementation Outcomes								Adaptations	Barriers/Facilitators
	Acceptability	Adoption	Appropriateness	Cost	Feasibility	Fidelity	Penetration	Sustainability		
McWilliams (2020)	R[3]	-	-	-	R[3]	-	-	-	-	R
Miller & Keating (2013)	-	-	-	-	R[3]	-	-	-	-	R
Miller et al. (2010)	R[1]	-	-	-	R[3]	-	-	-	-	R
Murry et al. (2018)	R[1]	-	-	-	-	R[3]	-	-	-	-
Myers et al. (2020)	-	-	-	-	-	-	-	-	-	R
Nelson et al. (2012)	-	-	-	-	-	R[1]	-	-	-	R
Nolan (2014)	-	-	-	-	-	-	-	-	-	R
Norton et al. (2021)	-	-	-	-	-	R[2]	-	-	R	R
O'Connor et al. (2020)	-	-	-	-	-	-	-	-	R	R
O'Donovan (2020)	-	-	-	-	-	-	-	-	R	R
Ofoha et al. (2018)	R[3]	-	-	-	-	R[3]	-	-	-	R
Ogden et al. (2005)	-	-	-	-	-	R[2]	-	R[3]	R	R
Ogden et al. (2009)	-	-	-	-	-	R[2]	-	R[3]	R	R
Oppenheim-Weller & Zeira (2018)	-	-	R[1]	-	-	-	-	-	R	R
Orte Socias et al. (2016)	-	-	-	-	-	-	-	-	-	R

Study	Implementation Outcomes								Adaptations	Barriers/Facilitators
	Acceptability	Adoption	Appropriateness	Cost	Feasibility	Fidelity	Penetration	Sustainability		
Osburn (2013)	R[3]	-	-	-	-	R[3]	-	-	-	R
Owens (2019)	-	-	R[2]	R[1]	-	R[2]	-	-	-	-
Parker et al. (2020)	R[2]	R[1]	R[1]	-	R[3]	R[3]	-	R[1]	R	R
Parra Cardona et al. (2012)	-	-	-	-	-	-	-	-	R	R
Patras & Klest (2016)	-	-	-	-	-	-	-	-	-	R
Patrick et al. (2008)	-	-	-	-	-	-	-	-	-	R
Pickard et al. (2016)	R[3]	-	-	-	-	-	-	-	-	R
Polaha et al. (2018)	-	-	-	-	-	-	R[3]	-	-	-
Porta et al. (2018)	-	-	-	-	-	-	-	-	R	R
Poulsen et al. (2010)	-	-	-	-	-	R[2]	-	-	R	R
Puffer et al. (2017)	R[3]	R[1]	-	-	-	-	-	-	R	-
Quetsch (2019)	R[3]	-	-	R[3]	-	R[2]	-	-	-	R
Ramos (2019)	-	-	-	-	-	R[3]	-	-	-	-
Romney et al. (2014)	-	-	-	R[1]	-	-	-	-	-	R
Roosa Ordway et al. (2018)	R[1]	-	-	-	-	R[2]	-	-	-	R

Study	Implementation Outcomes								Adaptations	Barriers/Facilitators
	Acceptability	Adoption	Appropriateness	Cost	Feasibility	Fidelity	Penetration	Sustainability		
Rudo-Stern (2019)	-	-	-	-	-	R[2]	-	-	-	-
Saldana et al. (2021)	-	-	-	R[2]	-	R[3]	-	-	-	-
Scudder & Herschell (2015)	-	-	-	-	-	-	-	-	-	R
Scudder et al. (2017)	-	-	-	-	-	R[2]	-	R[3]	-	R
Self-Brown et al. (2011)	R[1]	-	-	-	-	R[1]	-	-	R	R
Shapiro & Charest (2020)	-	-	-	-	-	R[1]	-	-	-	R
Shapiro et al. (2015)	R[3]	-	-	-	R[3]	R[1]	-	R[3]	R	R
Sigmarsdóttir & Guðmundsdóttir (2013)	-	-	-	-	-	R[3]	-	-	R	-
Sim et al. (2020)	R[3]	-	-	-	-	-	-	-	-	-
Skale et al. (2020)	-	-	-	-	-	R[2]	-	R[3]	R	R
Smith et al. (2015)	R[3]	R[1]	-	-	R[3]	R[3]	R[3]	-	R	R
Stern et al. (2008)	-	-	-	-	-	R[3]	-	-	-	R
Stokes (2014)	R[3]	-	-	-	-	R[3]	-	-	R	R

Study	Implementation Outcomes								Adaptations	Barriers/Facilitators
	Acceptability	Adoption	Appropriateness	Cost	Feasibility	Fidelity	Penetration	Sustainability		
Suchman et al. (2020)	R^1	-	-	-	R^3	-	-	-	R	R
Taylor et al. (2015)	-	-	-	-	-	R^3	-	-	-	-
Thomson et al. (2014)	-	-	-	-	-	R^1	-	-	-	R
Turner & Sanders (2006)	R^3	-	-	-	-	R^3	-	-	-	-
Weaver et al. (2011)	-	R^2	-	-	-	-	-	-	-	R
Webster-Stratton et al. (2014)	-	-	-	-	-	R^3	-	-	-	-
West et al. (2022)	R^3	-	R^3	-	R^3	-	-	-	-	R
Whitaker et al. (2012)	-	-	-	-	-	R^3	-	-	-	R
Whitaker et al. (2015)	-	-	-	-	-	-	-	-	-	R
Woodfield et al. (2021)	R^3	-	-	-	-	-	-	-	R	R
Yingling et al. (2020)	R^3	-	-	-	R^3	R^1	-	-	R	R
Zerón (2017)	-	-	-	-	-	-	-	-	R	R

Note. R = Reported by the study; R^1 = The study just mentioned the implementation outcome; R^2 = The study monitored the implementation outcome; R^3 = The study measured the implementation outcome (i.e., presented the results).

References of the Included Studies (*n* = 145)

Aalborg, A. E., Miller, B. A., Husson, G., Byrnes, H. F., Bauman, K. E., & Spoth, R. L. (2010). Implementation of adolescent family-based substance use prevention programmes in health care settings: Comparisons across conditions and programmes. *Health Education Journal*, *71*(1), 53–61. https://doi.org/10.1177/0017896910386209

Aarons, G. A., Miller, E. A., Green, A. E., Perrott, J. A., & Bradway, R. (2012). Adaptation happens: A qualitative case study of implementation of the Incredible Years evidence-based parent training programme in a residential substance abuse treatment programme. *Journal of Children's Services*, *7*(4), 233–245. https://doi.org/10.1108/17466661211286463

Abdala, I. G., Murta, S. G., Menezes, J. C. L. D., Nobre-Sandoval, L. D. A., Gomes, M. D. S. M., Duailibe, K. D., & Farias, D. A. (2020). Barriers and facilitators in the Strengthening Families Program (SFP 10–14) implementation process in Northeast Brazil: A retrospective qualitative study. *International Journal of Environmental Research and Public Health*, *17*(19), 6979. https://doi.org/10.3390/ijerph17196979

Akin, B. A., Brook, J., Byers, K. D., & Lloyd, M. H. (2016a). Worker perspectives from the front line: Implementation of evidence-based interventions in child welfare settings. *Journal of Child and Family Studies*, *25*(3), 870–882. https://doi.org/10.1007/s10826-015-0283-7

Akin, B. A., Brook, J., Lloyd, M. H., Bhattarai, J., Johnson-Motoyama, M., & Moses, M. (2016b). A study in contrasts: Supports and barriers to successful implementation of two evidence-based parenting interventions in child welfare. *Child Abuse & Neglect*, *57*, 30–40. https://doi.org/10.1016/j.chiabu.2016.06.002

Allchin, B., Weimand, B. M., O'Hanlon, B., & Goodyear, M. (2022). A sustainability model for family-focused practice in adult mental health services. *Frontiers in Psychiatry*, *12*, 761889. https://doi.org/10.3389/fpsyt.2021.761889

Álvarez, M., Padilla, S., & Máiquez, M. L. (2016). Home and group-based implementation of the "Growing Up Happily in the Family" program in at-risk psychosocial contexts. *Psychosocial Intervention*, *25*(2), 69–78. https://doi.org/10.1016/j.psi.2016.03.006

Amador Buenabad, N. G., Sánchez Ramos, R., Schwartz, S., Gutiérrez López, M. L., Díaz Juárez, A. D., Ortiz Gallegos, A. B., González Ortega, T. G., Vázquez Pérez, L., Medina-Mora Icaza, M. E., Domenech Rodríguez, M. M., & Villatoro Velázquez, J. A. (2020). Cluster randomized trial of a

multicomponent school-based program in Mexico to prevent behavioral problems and develop social skills in children. *Child & Youth Care Forum*, *49*(3), 343–364. https://doi.org/10.1007/s10566-019-09535-3

Amorós-Martí, P., Byrne, S., Mateos-Inchaurrondo, A., Vaquero-Tió, E., & Mundet-Bolós, A. (2016). "Learning together, growing with family": The implementation and evaluation of a family support programme. *Psychosocial Intervention*, *25*(2), 87–93. https://doi.org/10.1016/j.psi.2016.02.002

Arim, R. G., Guevremont, A., Dahinten, V. S., & Kohen, D. E. (2017). The use of administrative data to study the Triple P – Positive Parenting Program. *International Journal of Child, Youth and Family Studies*, *8*(3/4), 59. https://doi.org/10.18357/ijcyfs83/4201718001

Arruabarrena, I., de Paul, J., & Cañas, M. (2019). Implementation of an early preventive intervention programme for child neglect: SafeCare. *Psicothema*, *31(4)*, 443–449. https://doi.org/10.7334/psicothema2019.190

Asgary-Eden, V., & Lee, C. M. (2011). So now we've picked an evidence-based program, what's next? Perspectives of service providers and administrators. *Professional Psychology: Research and Practice*, *42*(2), 169–175. https://doi.org/10.1037/a0022745

Ballard, J., & Wieling, E. (2017). *Assessing the feasibility of implementing a parenting intervention with Karen refugees.* (Publication No. 10287020) [Doctoral dissertation, University of Minnesota]. ProQuest Dissertations and Theses Global.

Barnett, E. R., Rosenberg, H. J., Rosenberg, S. D., Osofsky, J. D., & Wolford, G. L. (2014). Dissemination and implementation of child-parent psychotherapy in rural public health agencies. *Child and Adolescent Mental Health*, *19*(3), 215-218. https://doi.org/10.1111/camh.12041

Barnett, M., Brookman-Frazee, L., Yu, S. H., Lind, T., Lui, J., Timmer, S., Boys, D., Urquiza, A., Innes-Gomberg, D., Quick-Abdullah, D., & Lau, A. S. (2021). Train-to-Sustain: Predictors of sustainment in a large-scale implementation of Parent–Child Interaction Therapy. *Evidence-Based Practice in Child and Adolescent Mental Health*, *6*(2), 262–276. https://doi.org/10.1080/23794925.2020.1855613

Bartley, L. (2017). *Making it happen: Understanding factors related to worker and organizational fidelity to Family Connections, a child maltreatment prevention program* (Publication No. 10277695) [Doctoral dissertation, Faculty of the Graduate School of the University of Maryland]. ProQuest

Dissertations and Theses Global.

Berkel, C., McBride Murry, V., Roulston, K. J., & Brody, G. H. (2013). Understanding the art and science of implementation in the SAAF efficacy trial. *Health Education*, *113*(4), 297–323. https://doi.org/10.1108/09654281311329240

Berkel, C., Sandler, I. N., Wolchik, S. A., Brown, C. H., Gallo, C. G., Chiapa, A., Mauricio, A. M., & Jones, S. (2018). "Home practice is the program": Parents' practice of program skills as predictors of outcomes in the New Beginnings Program effectiveness trial. *Prevention Science*, *19*(5), 663–673. https://doi.org/10.1007/s11121-016-0738-0

Bloomquist, M. L., Giovanelli, A., Benton, A., Piehler, T. F., Quevedo, K., & Oberstar, J. (2016). Implementation and evaluation of evidence-based psychotherapeutic practices for youth in a mental health organization. *Journal of Child and Family Studies*, *25*(11), 3278–3292. https://doi.org/10.1007/s10826-016-0479-5

Brabson, L. A. (2017). *Exploring staff turnover in a large-scale EBT implementation initiative* (Publication No. 10274031) [Master's thesis, Eberly College of Arts and Sciences at West Virginia University]. ProQuest Dissertations and Theses Global.

Brabson, L. A., Herschell, A. D., Snider, M. D. H., Jackson, C. B., Schaffner, K. F., Scudder, A. T., Kolko, D. J., & Mrozowski, S. J. (2021). Understanding the effectiveness of the cascading model to implement Parent-Child Interaction Therapy. *The Journal of Behavioral Health Services & Research*, *48*(3), 427–445. https://doi.org/10.1007/s11414-020-09732-2

Breitenstein, S. M., Fogg, L., Garvey, C., Hill, C., Resnick, B., & Gross, D. (2010). Measuring implementation fidelity in a community-based parenting intervention. *Nursing Research*, *59*(3), 158–165. https://doi.org/10.1097/NNR.0b013e3181dbb2e2

Breitkreuz, R., McConnell, D., Savage, A., & Hamilton, A. (2011). Integrating Triple P into existing family support services: A case study on program implementation. *Prevention Science*, *12*(4), 411–422. https://doi.org/10.1007/s11121-011-0233-6

Butler, J. (2019). *An exploration of parenting programmes: Staff and parent experiences of implementation* (Publication No. 28217662) [Doctoral dissertation, Faculty of Biology, Medicine and Health at the University of Manchester]. ProQuest Dissertations and Theses Global.

Cantu, A., Hill, L., & Becker, L. (2010). Implementation quality of a family-focused preventive

intervention in a community-based dissemination. *Journal of Children's Services*, *5*(4), 18–30. https://doi.org/10.5042/jcs.2010.0692

Chaffin, M., Hecht, D., Aarons, G., Fettes, D., Hurlburt, M., & Ledesma, K. (2016). EBT fidelity trajectories across training cohorts using the interagency collaborative team strategy. *Administration and Policy in Mental Health and Mental Health Services Research*, *43*(2), 144–156. https://doi.org/10.1007/s10488-015-0627-z

Charest, É., & Gagné, M.-H. (2019a). Measuring and predicting service providers' use of an evidence-based parenting program. *Administration and Policy in Mental Health and Mental Health Services Research*, *46*(4), 542–554. https://doi.org/10.1007/s10488-019-00934-1

Charest, É., & Gagné, M.-H. (2019b). Service providers' initial stance toward the adoption of an evidence-based parenting program. *Children and Youth Services Review*, *104*, 104410. https://doi.org/10.1016/j.childyouth.2019.104410

Chase, R. M., Carmody, K. A., Lent, M., Murphy, R., Amaya-Jackson, L., Wray, E., Ake, G. S., Sullivan, K., White, D., Gurwitch, R., & Murray, K. (2019). Disseminating parent-child interaction therapy through the learning collaborative model on the adoption and implementation of an evidence-based treatment. *Children and Youth Services Review*, *101*, 131–141. https://doi.org/10.1016/j.childyouth.2019.03.043

Chlebowski, C., Magaña, S., Wright, B., & Brookman-Frazee, L. (2018). Implementing an intervention to address challenging behaviors for autism spectrum disorder in publicly-funded mental health services: Therapist and parent perceptions of delivery with Latinx families. *Cultural Diversity and Ethnic Minority Psychology*, *24*(4), 552–563. https://doi.org/10.1037/cdp0000215

Christian, A. S., Niec, L. N., Acevedo-Polakovich, I. D., & Kassab, V. A. (2014). Dissemination of an evidence-based parenting program: Clinician perspectives on training and implementation. *Children and Youth Services Review*, *43*, 8–17. https://doi.org/10.1016/j.childyouth.2014.04.005

Clarke, S.-A., Calam, R., Morawska, A., & Sanders, M. (2013). Developing web-based Triple P 'Positive Parenting Programme' for families of children with asthma: Triple P for children with asthma. *Child: Care, Health and Development*, *40*(4), 492–497. https://doi.org/10.1111/cch.12073

Clay, A. L. (2018). *Population-level implementation of Triple P: Practitioner-reported organizational barriers and facilitators to use* [Doctoral dissertation, Graduate Faculty of North Carolina State University]. http://www.lib.ncsu.edu/resolver/1840.20/35061

Cluver, L., Meinck, F., Yakubovich, A., Doubt, J., Redfern, A., Ward, C., Salah, N., De Stone, S., Petersen, T., Mpimpilashe, P., Romero, R. H., Ncobo, L., Lachman, J., Tsoanyane, S., Shenderovich, Y., Loening, H., Byrne, J., Sherr, L., Kaplan, L., & Gardner, F. (2016). Reducing child abuse amongst adolescents in low- and middle-income countries: A pre-post trial in South Africa. *BMC Public Health*, *16*(1), 567. https://doi.org/10.1186/s12889-016-3262-z

Cooper, B. R., Shrestha, G., Hyman, L., & Hill, L. (2016). Adaptations in a community-based family intervention: Replication of two coding schemes. *The Journal of Primary Prevention*, *37*(1), 33–52. https://doi.org/10.1007/s10935-015-0413-4

Côté, M.-K., & Gagné, M.-H. (2020a). Changes in practitioners' attitudes, perceived training needs and self-efficacy over the implementation process of an evidence-based parenting program. *BMC Health Services Research*, *20*(1), 1092. https://doi.org/10.1186/s12913-020-05939-3

Côté, M.-K, & Gagné, M.-H (2020b). Diversity in practitioners' perspectives on the implementation of the evidence-based Triple P—Positive Parenting Program. *Journal of Community & Applied Social Psychology*, *30*(5), 480–493. https://doi.org/10.1002/casp.2458

Dababnah, S., & Parish, S. L. (2014). Incredible Years Program tailored to parents of preschoolers with autism: Pilot results. *Research on Social Work Practice*, *26*(4), 372–385. https://doi.org/10.1177/1049731514558004

Dawson-Squibb, J.-J., Davids, E. L., Chase, R., Puffer, E., Rasmussen, J. D. M., Franz, L., & De Vries, P. J. (2022). Bringing Parent–Child Interaction Therapy to South Africa: Barriers and facilitators and overall feasibility: First steps to implementation. *International Journal of Environmental Research and Public Health*, *19*(8), 4450. https://doi.org/10.3390/ijerph19084450

De Paul, J., Arruabarrena, I., & Indias, S. (2015). Implantación piloto de dos programas basados en la evidencia (SafeCare e Incredible Years) en los Servicios de Protección Infantil de Gipuzkoa (España) [Pilot Implementation of two evidence-based programs (SafeCare and Incredible Years) in child protection services in Gipuzkoa (Spain)]. *Psychosocial Intervention*, *24*(2), 105–120. https://doi.org/10.1016/j.psi.2015.07.001

Delawarde-Saïas, C., Gagné, M.-H., Brunson, L., & Drapeau, S. (2018). Implementing a multilevel prevention strategy under an intersectoral partnership: The case of the Triple P Program. *Children and Youth Services Review*, *88*, 170–179. https://doi.org/10.1016/j.childyouth.2018.03.009

Denard, C. (2017). *Child welfare caseworkers: A pivotal role in the uptake of evidence-based practices*. [Doctor dissertation, University of Pennsylvania]. https://repository.upenn.edu/edissertations/2253

Estrada, Y., Lee, T. K., Huang, S., Tapia, M. I., Velázquez, M.-R., Martinez, M. J., Pantin, H., Ocasio, M. A., Vidot, D. C., Molleda, L., Villamar, J., Stepanenko, B. A., Brown, C. H., & Prado, G. (2017). Parent-centered prevention of risky behaviors among Hispanic youths in Florida. *American Journal of Public Health*, *107*(4), 607–613. https://doi.org/10.2105/AJPH.2017.303653

Estrada, Y., Lee, T. K., Wagstaff, R., M. Rojas, L., Tapia, M. I., Velázquez, M. R., Sardinas, K., Pantin, H., Sutton, M. Y., & Prado, G. (2018). eHealth Familias Unidas: Efficacy trial of an evidence-based intervention adapted for use on the internet with Hispanic families. *Prevention Science*, *20*(1), 68–77. https://doi.org/10.1007/s11121-018-0905-6

Fang, Z., Lachman, J. M., Qiao, D., & Barlow, J. (2022). Controlled trial of a short-term intensive parent training program within the context of routine services for autistic children in China. *Psychosocial Intervention*, *31*(2), 121–131. https://doi.org/10.5093/pi2022a9

Fawley-King, K., Trask, E., E. Calderón, N., A. Aarons, G., & F. Garland, A. (2014). Implementation of an evidence-based parenting programme with a Latina population: Feasibility and preliminary outcomes. *Journal of Children's Services*, *9*(4), 295–306. https://doi.org/10.1108/JCS-04-2014-0024

Fowles, T. R., Masse, J. J., McGoron, L., Beveridge, R. M., Williamson, A. A., Smith, M. A., & Parrish, B. P. (2018). Home-based vs. clinic-based Parent–Child Interaction Therapy: Comparative effectiveness in the context of dissemination and implementation. *Journal of Child and Family Studies*, *27*(4), 1115–1129. https://doi.org/10.1007/s10826-017-0958-3

Frantz, I., Stemmler, M., Hahlweg, K., Plück, J., & Heinrichs, N. (2015). Experiences in disseminating evidence-based prevention programs in a real-world setting. *Prevention Science*, *16*(6), 789–800. https://doi.org/10.1007/s11121-015-0554-y

Gagnon, D. (2016). *Partnering with parents to promote high school graduation: An evidence-based program evaluation* [Master's thesis, University of Washington]. http://hdl.handle.net/1773/36446

Gallitto, E., Romano, E., & Drolet, M. (2018). Caregivers' perspectives on the SafeCare® programme:

Implementing an evidence-based intervention for child neglect. *Child & Family Social Work*, *23*(2), 307–315. https://doi.org/10.1111/cfs.12419

Garcia, A. R., Myers, C., Morones, S., Ohene, S., & Kim, M. (2020). "It starts from the top": Caseworkers, supervisors, and Triple P providers' perceptions of implementation processes and contexts. *Human Service Organizations: Management, Leadership & Governance*, *44*(3), 266–293. https://doi.org/10.1080/23303131.2020.1755759

Glazemakers, I. (2012). *A population health approach to parenting support: Disseminating the Triple P-Positive Parenting Program in the province of Antwerp* (Publication No. 3535436) [Doctoral dissertation, University of Antwerp]. ProQuest Dissertations and Theses Global.

Gomi, S. (2015). *Therapists' insights about the cultural adaptation of evidence-based parent training* (Publication No. 3745991) [Doctoral dissertation, Graduate Faculty of the University of Kansas]. ProQuest Dissertations and Theses Global.

Gordon, H. M., & Cooper, L. D. (2015). A case study of Parent–Child Interaction Therapy: Flexible client-centered adaptation of an EST. *Clinical Case Studies*, *15*(2), 126-142. https://doi.org/10.1177/1534650115603819

Guastaferro, K., Miller, K., Shanley Chatham, J. R., Whitaker, D. J., McGilly, K., & Lutzker, J. R. (2017). Systematic braiding of two evidence-based parent training programs: Qualitative results from the pilot phase. *Family & Community Health*, *40*(1), 88–97. https://doi.org/10.1097/FCH.0000000000000129

Haroz, E. E., Ingalls, A., Wadlin, J., Kee, C., Begay, M., Neault, N., & Barlow, A. (2019). Utilizing broad-based partnerships to design a precision approach to implementing evidence-based home visiting. *Journal of Community Psychology*, *48*(4), 1100–1113. https://doi.org/10.1002/jcop.22281

Heerman, W. J., Schludnt, D., Harris, D., Teeters, L., Apple, R., & Barkin, S. L. (2018). Scale-out of a community-based behavioral intervention for childhood obesity: Pilot implementation evaluation. *BMC Public Health*, *18*(1), 498. https://doi.org/10.1186/s12889-018-5403-z

Herschell, A. D., Shaffer, S. L., Wallace, N. M., Maise, A. A., Schaffner, K. F., McNeil, C. B., Dotson, P., & Johnson, V. J. (2021). Hybrid implementation effectiveness trial: Home-based intensive family coaching to improve outcomes for medicaid-enrolled preschoolers. *Evidence-Based Practice in Child and Adolescent Mental Health*, *6*(2), 246–261.

https://doi.org/10.1080/23794925.2021.1908191

Hickey, G., McGilloway, S., O'Brien, M., Leckey, Y., Devlin, M., & Donnelly, M. (2018). Strengthening stakeholder buy-in and engagement for successful exploration and installation: A case study of the development of an area-wide, evidence-based prevention and early intervention strategy. *Children and Youth Services Review*, *91*, 185–195. https://doi.org/10.1016/j.childyouth.2018.06.008

Hill, L. G., Maucione, K., & K. Hood, B. (2007). A focused approach to assessing program fidelity. *Prevention Science*, *8*(1), 25–34. https://doi.org/10.1007/s11121-006-0051-4

Hill, L. G., Cooper, B. R., & Parker, L. A. (2018). Qualitative comparative analysis: A mixed-method tool for complex implementation questions. *The Journal of Primary Prevention*, *40*(1), 69–87. https://doi.org/10.1007/s10935-019-00536-5

Hodge, L. M., Turner, K. M. T., Sanders, M. R., & Forster, M. (2017). Factors that influence evidence-based program sustainment for family support providers in child protection services in disadvantaged communities. *Child Abuse & Neglect*, *70*, 134–145. https://doi.org/10.1016/j.chiabu.2017.05.017

Huebner, R. A., Hall, M. T., Walton, M. T., Smead, E., Willauer, T., & Posze, L. (2021). The Sobriety Treatment and Recovery Teams program for families with parental substance use: Comparison of child welfare outcomes through 12 months post-intervention. *Child Abuse & Neglect*, *120*, 105260. https://doi.org/10.1016/j.chiabu.2021.105260

Ionutiu, R. D. (2016). The effectiveness of a positive parenting program: Results of a preliminary study in a Romanian sample. *Cognition, Brain, Behavior: An Interdisciplinary Journal*, *20*(3), 141–158.

Jackson, C. B., Herschell, A. D., Schaffner, K. F., Turiano, N. A., & McNeil, C. B. (2017). Training community-based clinicians in parent-child interaction therapy: The interaction between expert consultation and caseload. *Professional Psychology: Research and Practice*, *48*(6), 481–489. https://doi.org/10.1037/pro0000149

Kerns, S. E. U., McCormick, E., Negrete, A., Carey, C., Haaland, W., & Waller, S. (2017). Predicting post-training implementation of a parenting intervention. *Journal of Children's Services*, *12*(4), 302–315. https://doi.org/10.1108/JCS-04-2017-0015

Klest, S. K. (2014). Clustering practitioners within service organizations may improve implementation outcomes for evidence-based programs. *Zeitschrift Für Psychologie*, *222*(1), 30–36.

https://doi.org/10.1027/2151-2604/a000163

Kumpfer, K. L., Rie, J., & O'Driscoll, R. (2012). Effectiveness of a culturally adapted strengthening families program 12–16 years for high-risk Irish families. *Child & Youth Care Forum*, *41*(2), 173–195. https://doi.org/10.1007/s10566-011-9168-0

Kutash, K., Cross, B., Madias, A., Duchnowski, A. J., & Green, A. L. (2012). Description of a fidelity implementation system: An example from a community-based children's mental health program. *Journal of Child and Family Studies*, *21*(6), 1028–1040. https://doi.org/10.1007/s10826-012-9565-5

Lachman, J. M., Kelly, J., Cluver, L., Ward, C. L., Hutchings, J., & Gardner, F. (2016). Process evaluation of a parenting program for low-income families in South Africa. *Research on Social Work Practice*, *28*(2), 188–202. https://doi.org/10.1177/1049731516645665

Lanier, P., Kohl, P. L., Benz, J., Swinger, D., Moussette, P., & Drake, B. (2011). Parent–Child Interaction Therapy in a community setting: Examining outcomes, attrition, and treatment setting. *Research on Social Work Practice*, *21*(6), 689–698. https://doi.org/10.1177/1049731511406551

Lau, A. S., Fung, J. J., Ho, L. Y., Liu, L. L., & Gudiño, O. G. (2011). Parent training with high-risk immigrant Chinese families: A pilot group randomized trial yielding practice-based evidence. *Behavior Therapy*, *42*(3), 413–426. https://doi.org/10.1016/j.beth.2010.11.001

Leclair M., I.-A., Paquette, G., & Letarte, M.-J. (2017). La fidélité d'implantation d'un programme probant au-delà de son implantation initiale: L'exemple de Ces Années Incroyables en protection de l'enfance de 2003 à 2013 [The fidelity of implementation of a convincing program beyond its initial implementation: The example of the Incredible Years in child protection from 2003 to 2013]. *Canadian Journal of Program Evaluation*, *32*(1), 90–108. https://doi.org/10.3138/cjpe.31142

Lee, S. S., August, G. J., Bloomquist, M. L., Mathy, R., & Realmuto, G. M. (2006). Implementing an evidence-based preventive intervention in neighborhood family centers: Examination of perceived barriers to program participation. *The Journal of Primary Prevention*, *27*(6), 573–597. https://doi.org/10.1007/s10935-006-0060-x

Lengnick-Hall, R., Fenwick, K., Hurlburt, M. S., Green, A., Askew, R. A., & Aarons, G. A. (2019). Let's talk about adaptation! How individuals discuss adaptation during evidence-based practice implementation. *Journal of Children's Services*, *14*(4), 266–277. https://doi.org/10.1108/JCS-05-

2018-0010

Lent, J. (2022). *The impact of intensive, group-format Parent-Child Interaction Therapy on positive parenting skills and oppositional behavior of young children* (Publication No. 29255232) [Doctoral dissertation, Hofstra University]. ProQuest Dissertations and Theses Global.

Lewis, E. M., Feely, M., Seay, K. D., Fedoravicis, N., & Kohl, P. L. (2016). Child welfare involved parents and Pathways Triple P: Perceptions of program acceptability and appropriateness. *Journal of Child and Family Studies*, *25*(12), 3760–3770. https://doi.org/10.1007/s10826-016-0526-2

Liebsack, B. K. (2019). *The Parent-Child Interaction Therapy family experiences and feedback study: A follow-up examination of attrition in an evidence-based treatment in community settings statewide* (Publication No. 27527057) [Doctoral dissertation, Eberly College of Arts and Sciences at West Virginia University]. ProQuest Dissertations and Theses Global.

Lindsay, G., Strand, S., & Davis, H. (2011). A comparison of the effectiveness of three parenting programmes in improving parenting skills, parent mental wellbeing and children's behaviour when implemented on a large scale in community settings in 18 English local authorities: The parenting early intervention pathfinder (PEIP). *BMC Public Health*, *11*(1), 962. https://doi.org/10.1186/1471-2458-11-962

Love, S. M., Sanders, M. R., Turner, K. M. T., Maurange, M., Knott, T., Prinz, R., Metzler, C., & Ainsworth, A. T. (2016). Social media and gamification: Engaging vulnerable parents in an online evidence-based parenting program. *Child Abuse & Neglect*, *53*, 95–107. https://doi.org/10.1016/j.chiabu.2015.10.031

Lyon, A. R., & Budd, K. S. (2010). A community mental health implementation of Parent–Child Interaction Therapy (PCIT). *Journal of Child and Family Studies*, *19*(5), 654–668. https://doi.org/10.1007/s10826-010-9353-z

Lyons, E. R., Nekkanti, A. K., Funderburk, B. W., & Skowron, E. A. (2022). Parent–Child Interaction Therapy supports healthy eating behavior in child welfare-involved children. *International Journal of Environmental Research and Public Health*, *19*(17), 10535. https://doi.org/10.3390/ijerph191710535

Margolis, K. L. (2013). *A pilot feasibility and effectiveness trial of the Family Check-Up parenting intervention with Spanish preadolescents and their families: A cultural adaptation and feasibility*

study to enhance evidence-based intervention research in Spain (Publication No. 3589532) [Doctoral dissertation, Graduate School of the University of Oregon]. ProQuest Dissertations and Theses Global.

Mathias, K., Nayak, P., Singh, P., Pillai, P., & Goicolea, I. (2022). Is the Parwarish parenting intervention feasible and relevant for young people and parents in diverse settings in India? A mixed methods process evaluation. *BMJ Open*, *12*(2), e054553. https://doi.org/10.1136/bmjopen-2021-054553

Matos, M., Torres, R., Santiago, R., Jurado, M., & Rodriguez, I. (2006). Adaptation of Parent-Child Interaction Therapy for Puerto Rican families: A preliminary study. *Family Process*, *45*(2), 205–222. https://doi.org/10.1111/j.1545-5300.2006.00091.x

Matsumoto, Y., Sofronoff, K., & Sanders, M. R. (2007). The efficacy and acceptability of the Triple P-Positive Parenting Program with Japanese parents. *Behaviour Change*, *24*(4), 205–218. https://doi.org/10.1375/bech.24.4.205

Mauricio, A. M., Rudo-Stern, J., Dishion, T. J., Letham, K., & Lopez, M. (2019). Provider readiness and adaptations of competency drivers during scale-up of the Family Check-Up. *The Journal of Primary Prevention*, *40*(1), 51–68. https://doi.org/10.1007/s10935-018-00533-0

McCoy, A., Lachman, J. M., Ward, C. L., Tapanya, S., Poomchaichote, T., Kelly, J., Mukaka, M., Cheah, P. Y., & Gardner, F. (2021). Feasibility pilot of an adapted parenting program embedded within the Thai public health system. *BMC Public Health*, *21*(1), 1009. https://doi.org/10.1186/s12889-021-11081-4

McDonald, L., Coover, G., Sandler, J., Thao, T., & Shalhoub, H. (2012). Cultural adaptation of an evidence-based parenting programme with elders from South East Asia in the US: Co-producing Families and Schools Together – FAST. *Journal of Children's Services*, *7*(2), 113–127. https://doi.org/10.1108/17466661211238673

McWilliams, M. A. (2020). *Texting to Increase the Impact of Parenting (TIIP) program: Examining the effects on parent engagement in the Incredible Years® basic preschool parenting program* [Doctoral dissertation, University of North Carolina]. https://doi.org/10.17615/rgxd-sy28

Miller, F. K., & Keating, D. P. (2013). Implementing an evidence-based parent–child mental health program in a high-risk community. *Canadian Journal of Community Mental Health*, *32*(1), 139–153. https://doi.org/10.7870/cjcmh-2013-011

Miller, K. S., MaRwell, K. D., Fasula, A. M., Parker, J. T., Zackery, S., & Wyckoff, S. C. (2010). Pre-risk

HIV-prevention paradigm shift: The feasibility and acceptability of the Parents Matter! program in HIV risk communities. *Public Health Reports*, *125*(1_suppl), 38–46. https://doi.org/10.1177/00333549101250S106

Murry, V. M., Berkel, C., & Liu, N. (2018). The closing digital divide: Delivery modality and family attendance in the Pathways for African American Success (PAAS) Program. *Prevention Science*, *19*(5), 642–651. https://doi.org/10.1007/s11121-018-0863-z

Myers, C., Garcia, A., Beidas, R., Trinh, R., & Yang, Z. (2020). A theory of planned behavior exploration of child welfare caseworker referrals to an evidence-based parenting program. *Journal of Social Service Research*, *46*(6), 877–889. https://doi.org/10.1080/01488376.2019.1705458

Nelson, M. M., Shanley, J. R., Funderburk, B. W., & Bard, E. (2012). Therapists' attitudes toward evidence-based practices and implementation of Parent–Child Interaction Therapy. *Child Maltreatment*, *17*(1), 47–55. https://doi.org/10.1177/1077559512436674

Nolan, M. F. (2014). *Evaluation of the implementation of the Triple P Positive Parenting Program in PITT county* [Master's thesis, Faculty of the Department of Child Development and Family Relations East Carolina University]. http://hdl.handle.net/10342/4681

Norton, L., Hart, L. M., Butel, F., Moloney, S., O'Connor, N., Attenborough, V., & Roberts, S. (2021). Promoting Confident Body, Confident Child in community child health: A mixed-methods implementation study. *Health Promotion Journal of Australia*, *33*(1), 297–305. https://doi.org/10.1002/hpja.487

O'Connor, T. M., Perez, O., Beltran, A., Colón García, I., Arredondo, E., Parra Cardona, R., Cabrera, N., Thompson, D., Baranowski, T., & Morgan, P. J. (2020). Cultural adaptation of 'Healthy Dads, Healthy Kids' for Hispanic families: Applying the ecological validity model. *International Journal of Behavioral Nutrition and Physical Activity*, *17*(1), 52. https://doi.org/10.1186/s12966-020-00949-0

O'Donovan, L. (2020). *An exploration of the factors that influence practitioners' decision-making in the context of delivering the Strengthening Families Programme.* [Unpublished doctoral dissertation]. National University of Ireland, Cork.

Ofoha, D., Ogidan, R., & Saidu, R. (2018). Child discipline and violence in Nigeria: A community-based intervention programme to reduce violent discipline and other forms of negative parenting practices. *Review of Education*, *7*(3), 455–492. https://doi.org/10.1002/rev3.3128

Ogden, T., Amlund Hagen, K., Askeland, E., & Christensen, B. (2009). Implementing and evaluating evidence-based treatments of conduct problems in children and youth in Norway. *Research on Social Work Practice*, *19*(5), 582–591. https://doi.org/10.1177/1049731509335530

Ogden, T., Forgatch, M. S., Askeland, E., Patterson, G. R., & Bullock, B. M. (2005). Implementation of parent management training at the national level: The case of Norway. *Journal of Social Work Practice*, *19*(3), 317–329. https://doi.org/10.1080/02650530500291518

Oppenheim-Weller, S., & Zeira, A. (2018). SafeCare in Israel: The challenges of implementing an evidence-based program. *Children and Youth Services Review*, *85*, 187–193. https://doi.org/10.1016/j.childyouth.2017.12.031

Orte Socias, C., Ballester Brage, L., Pascual Barrio, B., Gomila Grau, M. A., & Amer Fernández, J. (2016). Las competencias de los formadores en el Programa de Competencia Familiar, un programa de educación familiar basado en la evidencia [The competencies of the trainers in the Family Competence Program, an evidence-based family education program]. *Revista Complutense de Educación*, *29*(3), 651–663. https://doi.org/10.5209/RCED.53547

Osburn, J. L. (2013). *An investigation of the effectiveness and transportability of the Incredible Years self-administered parent training program with an at-risk Head Start sample* (Publication No. 3593476) [Doctoral dissertation, Michigan State University]. ProQuest Dissertations and Theses Global.

Owens, C. R. (2019). *The role of peer assisted supervision and support in providers' use of Triple P-Positive Parenting Program* (Publication No. 27732069) [Master's thesis, Graduate Faculty of North Carolina State University]. ProQuest Dissertations and Theses Global.

Parker, A., Johnson-Motoyama, M., Mariscal, E. S., Guilamo-Ramos, V., Reynoso, E., & Fernandez, C. (2020). Novel service delivery approach to address reproductive health disparities within immigrant Latino communities in geographic hot spots: An implementation study. *Health & Social Work*, *45*(3), 155–163. https://doi.org/10.1093/hsw/hlaa014

Parra Cardona, J. R., Domenech-Rodriguez, M., Forgatch, M., Sullivan, C., Bybee, D., Holtrop, K., Escobar-Chew, A. R., Tams, L., Dates, B., & Bernal, G. (2012). Culturally adapting an evidence-based parenting intervention for Latino immigrants: The need to integrate fidelity and cultural relevance. *Family Process*, *51*(1), 56–72. https://doi.org/10.1111/j.1545-5300.2012.01386.x

Patras, J., & Klest, S. (2016). Group size and therapists' workplace ratings: Three is the magic number. *Journal of Social Work*, *16*(2), 216–227. https://doi.org/10.1177/1468017315581564

Patrick, M. E., Rhoades, B. L., Small, M., & Coatsworth, J. D. (2008). Faith-placed parenting intervention. *Journal of Community Psychology*, *36*(1), 74–80. https://doi.org/10.1002/jcop.20218

Pickard, K. E., Wainer, A. L., Bailey, K. M., & Ingersoll, B. R. (2016). A mixed-method evaluation of the feasibility and acceptability of a telehealth-based parent-mediated intervention for children with autism spectrum disorder. *Autism*, *20*(7), 845–855. https://doi.org/10.1177/1362361315614496

Polaha, J., Schetzina, K. E., Baker, K., & Morelen, D. (2018). Adoption and reach of behavioral health services for behavior problems in pediatric primary care. *Families, Systems, & Health*, *36*(4), 507–512. https://doi.org/10.1037/fsh0000380

Porta, C. M., Bloomquist, M. L., Garcia-Huidobro, D., Gutiérrez, R., Vega, L., Balch, R., Yu, R., & Cooper, D. K. (2018). Bi-national cross-validation of an evidence-based conduct problem prevention model. *Cultural Diversity and Ethnic Minority Psychology*, *24*(2), 231–241. https://doi.org/10.1037/cdp0000178

Poulsen, M. N., Vandenhoudt, H., Wyckoff, S. C., Obong'o, C. O., Ochura, J., Njika, G., Otwoma, N. J., & Miller, K. S. (2010). Cultural adaptation of a U.S. evidence-based parenting intervention for rural Western Kenya: From Parents Matter! to Families Matter! *AIDS Education and Prevention: Official Publication of the International Society for AIDS Education*, *22*(4), 273–285. https://doi.org/10.1521/aeap.2010.22.4.273

Puffer, E. S., Annan, J., Sim, A. L., Salhi, C., & Betancourt, T. S. (2017). The impact of a family skills training intervention among Burmese migrant families in Thailand: A randomized controlled trial. *PLOS ONE*, *12*(3), e0172611. https://doi.org/10.1371/journal.pone.0172611

Quetsch, L. B. (2019). *A randomized controlled trial of Parent-Child Interaction Therapy with and without incentives in a community mental health setting* [Doctoral dissertation, Eberly College of Arts and Sciences

at West Virginia University]. https://doi.org/10.33915/etd.6463

Ramos, G. G. (2019). *Helping mothers help their children cope with stress: Evaluation of a program for Latina mothers* [Doctoral dissertation, Washington State University]. https://hdl.handle.net/2376/17901

Romney, S., Israel, N., & Zlatevski, D. (2014). Exploration-stage implementation variation: Its effect on the cost-effectiveness of an evidence-based parenting program. *Zeitschrift Für Psychologie*, *222*(1), 37–48. https://doi.org/10.1027/2151-2604/a000164

Roosa Ordway, M., McMahon, T. J., De Las Heras Kuhn, L., & Suchman, N. E. (2018). Implementation of an evidenced-based parenting program in a community mental health setting. *Infant Mental Health Journal*, *39*(1), 92–105. https://doi.org/10.1002/imhj.21691

Rudo-Stern, J. (2019). *Comparison of video and audio rating modalities for assessment of provider fidelity to a family-centered, evidence-based program* [Doctoral dissertation, Arizona State University]. https://keep.lib.asu.edu/items/158498

Saldana, L., Chapman, J. E., Campbell, M., Alley, Z., Schaper, H., & Padgett, C. (2021). Meeting the needs of families involved in the child welfare system for parental substance abuse: Outcomes from an effectiveness trial of the Families Actively Improving Relationships program. *Frontiers in Psychology*, *12*, 689483. https://doi.org/10.3389/fpsyg.2021.689483

Scudder, A. T., & Herschell, A. D. (2015). Building an evidence-base for the training of evidence-based treatments in community settings: Use of an expert-informed approach. *Children and Youth Services Review*, *55*, 84–92. https://doi.org/10.1016/j.childyouth.2015.05.003

Scudder, A. T., Taber-Thomas, S. M., Schaffner, K., Pemberton, J. R., Hunter, L., & Herschell, A. D. (2017). A mixed-methods study of system-level sustainability of evidence-based practices in 12 large-scale implementation initiatives. *Health Research Policy and Systems*, *15*(1), 102. https://doi.org/10.1186/s12961-017-0230-8

Self-Brown, S., Frederick, K., Binder, S., Whitaker, D., Lutzker, J., Edwards, A., & Blankenship, J. (2011). Examining the need for cultural adaptations to an evidence-based parent training program targeting the prevention of child maltreatment. *Children and Youth Services Review*, *33*(7), 1166–1172. https://doi.org/10.1016/j.childyouth.2011.02.010

Shapiro, C. J., & Charest, E. (2020). Factors associated with provider self-efficacy in delivery of evidence-based programs for children, youth, and families. *Child & Family Social Work*, *25*(3), 637–647. https://doi.org/10.1111/cfs.12738

Shapiro, C. J., Prinz, R. J., & Sanders, M. R. (2015). Sustaining use of an evidence-based parenting intervention: Practitioner perspectives. *Journal of Child and Family Studies*, *24*(6), 1615–1624. https://doi.org/10.1007/s10826-014-9965-9

Sigmarsdóttir, M., & Guðmundsdóttir, E. V. (2013). Implementation of Parent Management Training-Oregon Model (PMTO^{TM}) in Iceland: Building sustained fidelity. *Family Process*, *52*(2), 216–227. https://doi.org/10.1111/j.1545-5300.2012.01421.R

Sim, A. L., Bowes, L., Maignant, S., Magber, S., & Gardner, F. (2021). Acceptability and preliminary outcomes of a parenting intervention for Syrian refugees. *Research on Social Work Practice*, *31*(1), 14–25. https://doi.org/10.1177/1049731520953627

Skale, G., Perez, H., & Williams, M. E. (2020). Factors influencing implementation of evidence-based mental health interventions for infants and young children. *The Journal of Behavioral Health Services & Research*, *47*(4), 493–508. https://doi.org/10.1007/s11414-020-09694-5

Smith, J. D., Stormshak, E. A., & Kavanagh, K. (2015). Results of a pragmatic effectiveness–implementation hybrid trial of the Family Check-Up in community mental health agencies. *Administration and Policy in Mental Health and Mental Health Services Research*, *42*(3), 265–278. https://doi.org/10.1007/s10488-014-0566-0

Stern, S. B., Alaggia, R., Watson, K., & Morton, T. R. (2008). Implementing an evidence-based parenting program with adherence in the real world of community practice. *Research on Social Work Practice*, *18*(6), 543–554. https://doi.org/10.1177/1049731507308999

Stokes, J. (2014). *Effectiveness of community-delivered Parent-Child Interaction Therapy compared to treatment as usual* [Doctoral dissertation, West Virginia University]. https://researchrepository.wvu.edu/etd/197

Suchman, N., Berg, A., Abrahams, L., Abrahams, T., Adams, A., Cowley, B., Decoste, C., Hawa, W., Lachman, A., Mpinda, B., Cader-Mokoa, N., Nama, N., & Voges, J. (2020). Mothering from the Inside Out: Adapting an evidence-based intervention for high-risk mothers in the Western Cape of South Africa. *Development and Psychopathology*, *32*(1), 105–122. https://doi.org/10.1017/S0954579418001451

Taylor, W. D., Asgary-Eden, V., Lee, C. M., & LaRoche, K. J. (2015). Service providers' adherence to an evidence-based parenting program: What are they missing and why? *Journal of Child and Family Studies*, *24*(1), 50–56. https://doi.org/10.1007/s10826-013-9812-4

Thomson, S., Michelson, D., & Day, C. (2014). From parent to 'peer facilitator': A qualitative study of a peer-led parenting programme. *Child: Care, Health and Development*, *41*(1), 76–83. https://doi.org/10.1111/cch.12132

Turner, K. M. T., & Sanders, M. R. (2006). Help when it's needed first: A controlled evaluation of brief, preventive behavioral family intervention in a primary care setting. *Behavior Therapy*, *37*(2), 131–142. https://doi.org/10.1016/j.beth.2005.05.004

Weaver, N. L., Nansel, T. R., Williams, J., Tse, J., Botello-Harbaum, M., & Willson, K. (2011). Reach of a kiosk-based pediatric injury prevention program. *Translational Behavioral Medicine*, *1*(4), 515–522. https://doi.org/10.1007/s13142-011-0066-7

Webster-Stratton, C. H., Reid, M. J., & Marsenich, L. (2014). Improving therapist fidelity during implementation of evidence-based practices: Incredible Years program. *Psychiatric Services*, *65*(6), 789–795. https://doi.org/10.1176/appi.ps.201200177

West, A. L., Berlin, L. J., Goodman, A., Endy, K., Manzon, C., & Harden, B. J. (2022). Home-based Early Head Start plus Attachment and Biobehavioral Catch-up: A qualitative study of implementation outcomes. *Journal of Child and Family Studies*, *31*(4), 1057–1068. https://doi.org/10.1007/s10826-021-02189-7

Whitaker, D. J., Rogers-Brown, J. S., Cowart-Osborne, M., Self-Brown, S., & Lutzker, J. R. (2015). Public child welfare staff knowledge, attitudes, and referral behaviors for an evidence-based parenting program. *Psychosocial Intervention*, *24*(2), 89–95. https://doi.org/10.1016/j.psi.2015.06.001

Whitaker, D. J., Ryan, K. A., Wild, R. C., Self-Brown, S., Lutzker, J. R., Shanley, J. R., Edwards, A. M., McFry, E. A., Moseley, C. N., & Hodges, A. E. (2012). Initial implementation indicators from a statewide rollout of SafeCare within a child welfare system. *Child Maltreatment*, *17*(1), 96–101. https://doi.org/10.1177/1077559511430722

Woodfield, M. J., Cargo, T., Merry, S. N., & Hetrick, S. E. (2021). Barriers to clinician implementation of Parent-Child Interaction Therapy (PCIT) in New Zealand and Australia: What role for time-out? *International Journal of Environmental Research and Public Health*, *18*(24), 13116. https://doi.org/10.3390/ijerph182413116

Yingling, M. E., Hock, R. M., Feinberg, M. E., & Holbert, A. A. (2020). Community-engaged process to adapt evidence-based programs for parents of children with autism spectrum disorder. *Children and Youth Services Review*, *112*, 104876. https://doi.org/10.1016/j.childyouth.2020.104876

Zerón, G. A. L. (2017). *Exploring the relevance of a culturally adapted parenting intervention for low-income ethnic minority families involved in the child welfare system: A qualitative study with*

parents and interventionists (Publication No. 10686853) [Doctoral dissertation, Michigan State University]. ProQuest Dissertations and Theses Global.
